# Serial transplantation unmasks galectin-9 contribution to tumor immune escape in the MB49 murine model

**DOI:** 10.1038/s41598-021-84270-1

**Published:** 2021-03-04

**Authors:** Valentin Baloche, Julie Rivière, Thi Bao Tram Tran, Aurore Gelin, Olivia Bawa, Nicolas Signolle, M′Boyba Khadija Diop, Philippe Dessen, Stéphanie Beq, Muriel David, Pierre Busson

**Affiliations:** 1 CNRS, UMR 9018, Gustave Roussy and Université Paris-Saclay, 39 Rue Camille Desmoulins, F-94805 Villejuif, France; 2 Inserm, U1170, Gustave Roussy, 39 Rue Camille Desmoulins, F-94805 Villejuif, France; 3 Plateforme de pathologie expérimentale et translationnelle, UMS AMMICA, Gustave Roussy, 39 Rue Camille Desmoulins, F-94805 Villejuif, France; 4 Plateforme de Bioinformatique, UMS AMMICA, Gustave Roussy, 39 Rue Camille Desmoulins, F-94805 Villejuif, France; 5 HiFiBiO Therapeutics, Pépinière Paris Santé Cochin, 29 Rue du Faubourg Saint-Jacques, F-75014 Paris, France

**Keywords:** Cancer microenvironment, Cancer models, Tumour immunology, Bladder cancer

## Abstract

Mechanisms of tumor immune escape are quite diverse and require specific approaches for their exploration in syngeneic tumor models. In several human malignancies, galectin-9 (gal-9) is suspected to contribute to the immune escape. However, in contrast with what has been done for the infiltrating cells, the contribution of gal-9 produced by malignant cells has never been demonstrated in an animal model. Therefore, we derived isogenic clones—either positive or negative for gal-9—from the MB49 murine bladder carcinoma cell line. A progressive and consistent reduction of tumor growth was observed when gal-9-KO cells were subjected to serial transplantations into syngeneic mice. In contrast, tumor growth was unaffected during parallel serial transplantations into nude mice, thus linking tumor inhibition to the enhancement of the immune response against gal-9-KO tumors. This stronger immune response was at least in part explained by changing patterns of response to interferon-γ. One consistent change was a more abundant production of CXCL10, a major inflammatory factor whose production is often induced by interferon-γ. Overall, these observations demonstrate for the first time that serial transplantation into syngeneic mice can be a valuable experimental approach for the exploration of novel mechanisms of tumor immune escape.

## Introduction

Galectins are a family of mammalian lectins sharing the capacity to bind β-galactoside bonds contained in glycans carried by glycolipids or glycoproteins^1^. Each galectin selectively binds a specific range of glycans which can be located inside the cells, at the cell surface or in the extra-cellular matrix. Several galectins can be released in the extra-cellular medium by non-classical secretory pathways and behave like intercellular messengers. Galectin-9 (gal-9) plays a major role in the physiology of the immune system^2^. When secreted in the extra-cellular medium, it behaves somehow like a cytokine with mainly immunosuppressive effects. However, it has a broader range of potential surface receptors than most *bona fide* cytokines. Its effects are dependent on multiple surface glycoproteins or glycolipids containing β-galactoside bonds, for example Tim-3 (on T-cells) and Dectin-1 (on macrophages)^3,4^. In physiological conditions, gal-9 is expressed at a low level in most tissues and organs. Its production is enhanced under inflammatory conditions. This occurs in epithelial and endothelial cells, under stimulation by interferon-γ^4^.

Several years ago, our group was the first to show the contribution of gal-9 to immune suppression in the micro- and macro-environment of a human malignancy, namely nasopharyngeal carcinoma, a tumor which is almost constantly associated with the Epstein-Barr virus (EBV)^5^. Since this publication, the contribution of gal-9 to immune evasion has been reported in a number of other malignancies as diverse as lung, breast and pancreatic carcinomas, melanomas and acute myeloid leukemias^3,6–9^. In vitro and in animal models, the immunosuppressive effects of gal-9 result from the inhibition of both the innate and adaptative immune responses^10^. Regarding innate immunity, gal-9 inhibits NK cell cytotoxicity while enhancing the activity of MDSCs^11–13^. Regarding adaptive immunity, it is known to induce cell death of CD4^+^ Th1 and Th17 cells and exhaustion of CD8^+^ cells while enhancing the expansion and activity of suppressive cells like iTregs^4,14–17^. Despite multiple observations supporting the contribution of extracellular gal-9 to tumor immune escape, there are still many unknowns and controversies about the overall contribution of gal-9 to the malignant process. Difficulties and sometimes confusion in this research area are, in our view, due to two main causes: (1) cells producing gal-9 in the tumor microenvironment are quite diverse depending on the type of malignancies; (2) there is a suspicion that cell-associated gal-9—inside malignant cells or at their surface—can have anti-metastatic effects. This hypothesis is mainly supported by observations from a prospective cohort of breast carcinoma patients where the abundance of gal-9 in tumor tissue sections is inversely correlated to the risk of metastatic relapse^18^. On the other hand, elevated concentrations of plasma or serum gal-9 are consistently associated with a more severe prognosis in various types of malignancies, including pancreatic carcinomas, metastatic melanomas and renal carcinomas^6,19,20^.

In humans as well as in animal models, tumor gal-9 is often produced both by malignant cells and by infiltrating cells of lymphoid or myeloid origin^3,21^. So far, to our knowledge, there is no murine tumor model designed to assess the immunosuppressive effect of gal-9 produced by the malignant cells themselves separately from gal-9 released by the infiltrating cells. To address this need, we used a gene editing approach to create isogenic gal-9-positive and gal-9-negative clones derived from the murine bladder carcinoma cell line MB49. The MB49 line was chosen for 2 reasons: (1) it has a substantial baseline expression of gal-9; (2) it is known to trigger a robust although ineffective immune response in C57BL/6 syngeneic mice^22–24^. Using this approach, we could demonstrate that gal-9 gene ablation makes MB49 cells much more sensitive to the anti-tumor immune response. However, the emergence of this immune response was progressive, requiring at least 3 serial passages and growth cycles on syngeneic mice to reach full efficiency and blockade of tumor growth. To elucidate the mechanisms of the immune response promoted by gal-9 gene ablation, we combined detection of infiltrating leucocytes by IHC, assessment of the diversity of the stromal T-cell repertoire, differential RNAseq of gal-9-KO and WT tumors and intra-tumoral cytokine detection at successive cycles of tumor growth. One of the main changes occurring prior to the blockade of tumor growth was a broad increase in transcription of genes encoding proteins involved in tumor cell response to interferon-γ. Consistently, in vitro, the production of CXCL10 induced by exogenous interferon-γ was more abundant in gal-9-KO cells than in control clones. *A contrario*, a poor response of gal-9-positive malignant cells to interferon-γ might be one mechanism supporting the immune escape of wild-type MB49 tumors.

## Results

### Genetic ablation of the gal-9 gene in the MB49 cells and in vitro characterization of the gal-9-KO and control clones

In order to explore the contribution of gal-9 produced by malignant cells to tumor immune escape, we selected the MB49 murine tumor model. MB49 cells originated from a bladder carcinoma and are compatible with C57BL/6 mice. They are known for generating hot tumors in syngeneic mice and to trigger relatively strong although inefficient primarily CD8 cytolytic adaptive immune response^22–24^. They are also known to be responsive to immune-checkpoint inhibitors. MB49 cells were shown to have constitutive, permanent expression of gal-9. The *Lgals9* gene was invalidated in clones derived from MB49 cells using the CRISPR/Cas9 strategy (Supplementary Figure S1A). This procedure resulted in three clones knocked-out for gal-9 (gal-9-KO clones: # 175, 345, 377) and 2 control clones (WT-Ctrl clones: # 116 and 376) which had undergone the same transfection and cloning process but had retained an intact *Lgals9* gene and gal-9 expression similar to the parental MB49 cells (Supplementary Figure S1B,C). We first observed that cell proliferation in vitro was not affected in gal-9-KO clones by comparison with WT-Ctrl clones and parental MB49 (Supplementary Figure S2). Comparative RNAseq analysis of gal-9-KO and control MB49 cells propagated in vitro resulted in two important observations: (1) the absence of a gene subset with a distinct pattern of expression in gal-9-KO by comparison with WT-Ctrl clones; (2) the absence of distinct transcriptional alterations for genes predicted to be vulnerable to off-target effects in gal-9-KO by comparison with WT-Ctrl clones (Supplementary Figures S3 and S4). Finally, we found no influence of gal-9 silencing on the expression of other galectins: gal-1, -3 and -8 (Supplementary Figure S5).

### A serial tumor transplantation assay unmasks the immune restriction of tumor growth for gal-9-KO MB49 cells

**Figure 1:**
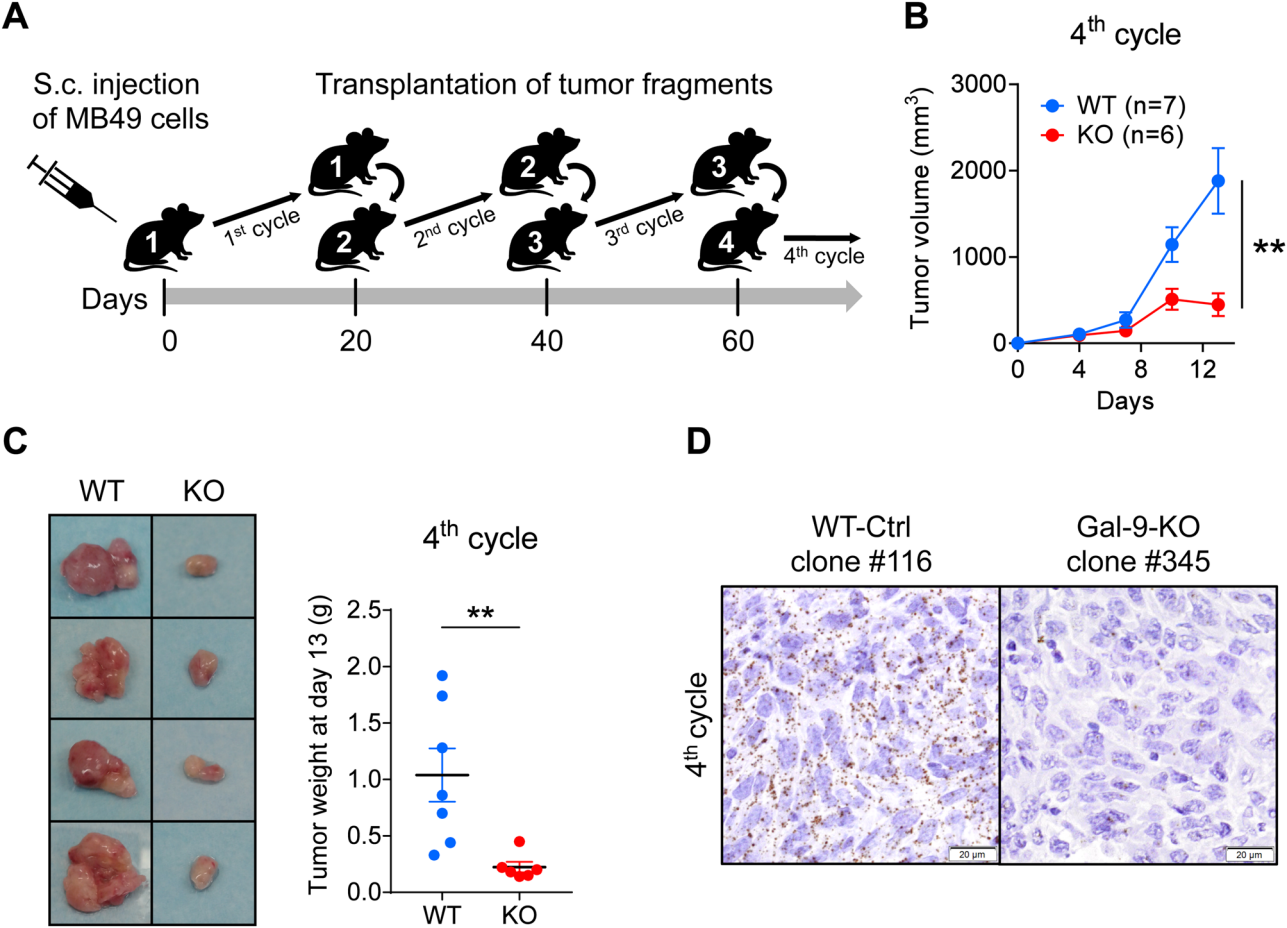
Pilot serial tumor transplantation assay using MB49 cells and isogenic clones in syngeneic mice (C57BL/6 N). (**A**) Cartoon depicting the main steps of the assay. The MB49 parental cell line (n = 3), the WT-Ctrl clone #116 (n = 4), the gal-9-KO clones #345 (n = 2) and #377 (n = 4), were subcutaneously injected into C57BL/6 female mice (opening the 1st cycle of tumor growth involving mice n°1). Twenty days later, tumors were collected and cut into small fragments. For each mouse donor, a small aggregate of tumor fragments (100 mg) was then subcutaneously transplanted into a single recipient mouse (2nd cycle involving mice n°2). This operation was repeated twice (opening the 3rd and 4th cycles of tumor growth involving mice n°3 and mice n°4 respectively). (**B**) Comparison of tumor growth curves for WT and KO tumors during the 4th cycle (mean volumes ± SEM). WT tumors include tumors derived from the parental cells (MB49) and the WT-Ctrl clone #116. Significant differences in tumor volumes were recorded at day 13 (Mann–Whitney test). (**C**) Collection and comparative inspection of WT and gal-9-KO tumors at the completion of the 4th cycle of tumor growth. *Left panel:* visualization of selected representative tumors (Ctrl clone #116 and KO clone #377). The growth of gal-9-KO tumors was drastically reduced. Only small necrotic tumors were recovered. *Right panel:* graph showing a comparison of tumor weights for all mice of the 2 groups (7 and 6 mice respectively, Mann–Whitney test). (**D**) RNAscope detection of gal-9 transcripts in WT and KO tumor sections at the completion of the 4th cycle of tumor growth. Several WT and KO tumors were fixed in paraformaldehyde and paraffin-embedded. Then, tumor sections were subjected to in situ hybridization of gal-9 mRNA using the RNAscope method according to the manufacturer instructions (ACD—Biotechne). In tumors derived from parental and WT-Ctrl cells, numerous dots corresponding to gal-9 transcripts were detected in the vast majority of the cells. On the contrary, in gal-9-KO tumors, gal-9 RNA dots were much less abundant and restricted to some isolated cells (likely infiltrating immune cells or endothelial cells from the recipient mice).

**Figure 2:**
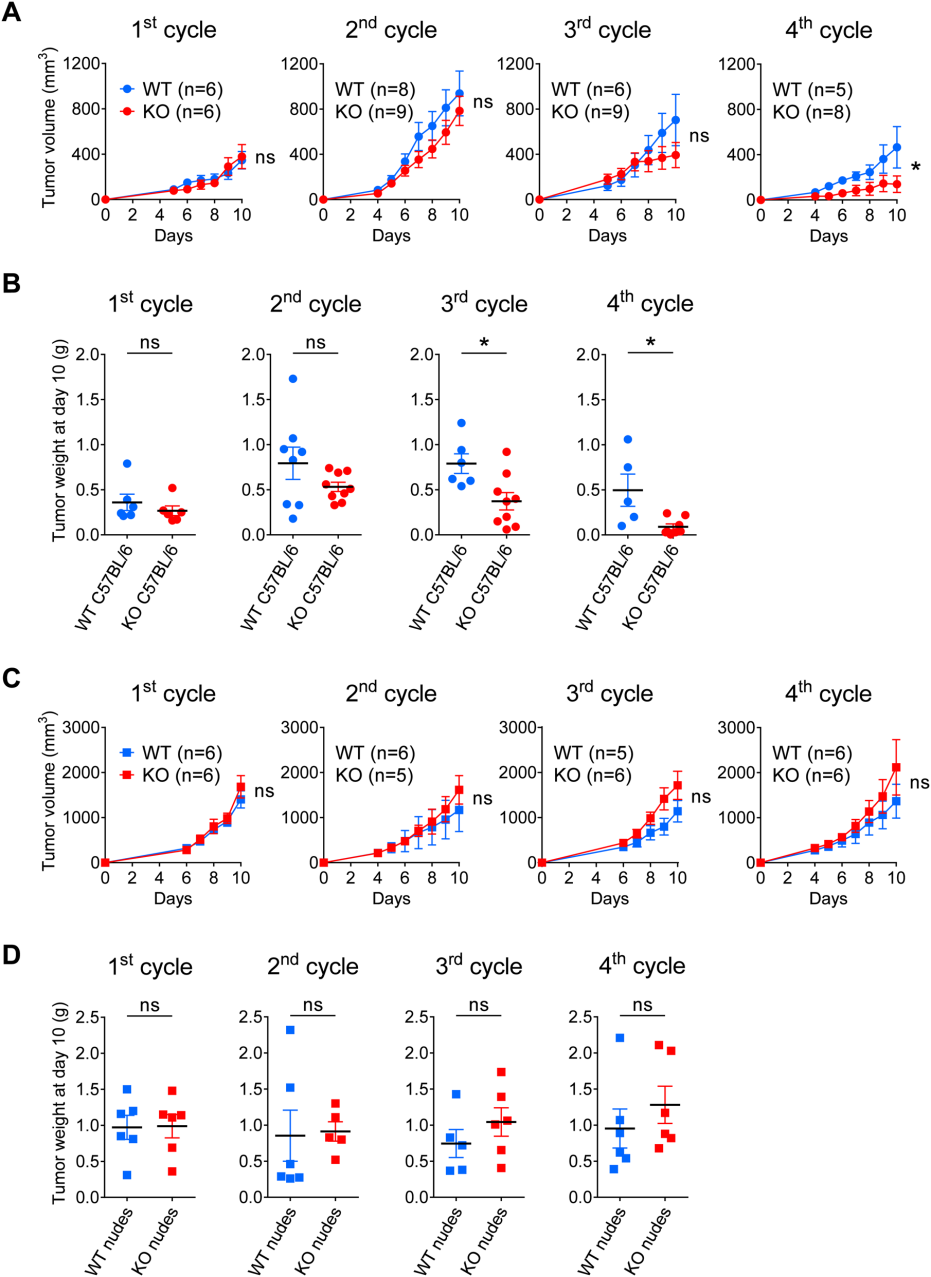
Serial tumor transplantation assays of MB49 cells and isogenic clones using immunocompetent or immunodeficient mice. (**A**) Comparison of tumor growth curves for WT and KO tumors at each cycle (mean volumes ± SEM). WT tumors include tumors derived from the parental cells (MB49) and the 2 WT-Ctrl clones # 116 and 376. For the 1st cycle of tumor growth, cells from the MB49 parental cell line (n = 3), WT-Ctrl clones # 116 (n = 3), 376 (n = 3) and gal-9-KO clones # 175 (n = 3), 345 (n = 3) and 377 (n = 3), were subcutaneously injected into C57BL/6N female mice. Subsequent passages were done using subcutaneous inoculation of aggregated tumor fragments (70 mg). In most cases, tumor fragments collected from one animal were inoculated in a single recipient mouse. Thus, we had 2 or 3 tumor lineages for each clone and for MB49 parental cells. Comparison of WT and KO groups were performed at the last point of each cycle and subjected to Mann–Whitney tests. As in the pilot experiment, the serial transplantations into syngeneic mice led to a progressive decrease in tumor volume for gal-9-KO tumors compared to WT-Ctrl and parental MB49-derived tumors. (**B**) Comparison of the tumor weights when mice were sacrificed at the completion of each cycle each ten days. The differences between the two groups were significant from the 3rd cycle (Mann–Whitney test). (**C** and **D**) The same experimental scheme was carried out in parallel using nude mice. In the context of these immunodeficient mice, there was no growth reduction during serial transplantation for gal-9-KO-derived tumors, as compared to WT-derived tumors (a slight, though non-significant, increase was actually observed). These observations highlight the contribution of the adaptative immune response to the reduction of gal-9-KO tumor growth.

Moving from in vitro to in vivo experiments, the next step of our study was to investigate the growth of tumors resulting from subcutaneous injections of gal-9-KO or control MB49 cells. However, we often faced technical concerns regarding the mode of injections of these cells: either early skin ulcerations resulting from superficial injections or unwanted infiltration of underlying organs when making deeper injections. This prompted us to set up tumor growth assays based on subcutaneous implantation of small tumor fragments. Cell injection was the method retained for the first passage allowing collection of tumor fragments for subsequent passages. This approach based on the implantation of small tumor fragments was successful in reducing the incidence of tumor ulcerations or tumor infiltrations in underlying organs. In addition, from multiple preliminary experiments like the one depicted in Fig. 1, progressively emerged the evidence that tumor growth of gal-9-KO clones was undergoing a progressive decline after several passages on syngeneic mice (C57BL/6 N) when using small tumor fragments in serial transplantations. This experimental sequence was thereafter designated as “serial tumor transplantation assay”. Using a variant of the protocol used for Fig. 1, we designed a larger experiment involving all 3 gal-9-KO clones along with the two WT-Ctrl clones and parental MB49 cells, with concomitant transplantation on immune-competent syngeneic (C57BL/6 N) and immune-deficient mice (Supplementary Figure S6). As shown in Fig. 2A, a progressive reduction of tumor growth was observed for gal-9-KO clones through successive passages. This reduction was hardly visible at the 2nd cycle of tumor growth, but stronger at the 3rd cycle although not statistically significant. At the 4th cycle, it was even more marked and statistically significant (individual growth curves are visible in Supplementary Figure S7A,B). Data based on tumor growth curves were confirmed by weight measurements of tumors excised following mouse euthanasia at the completion of each cycle, every 10 days. As shown in panel B of Fig. 2, the relative decline of tumor growth for gal-9-KO clones transplanted on syngeneic mice was statistically significant at cycle 3 and even more dramatic at cycle 4. Beyond cycle 4, we made two additional graft cycles involving all control tumors (n = 5) along with remaining gal-9-KO tumors (those weighting more than 90 mg at the completion of the 4th cycle, n = 3). At these next steps, all but one gal-9-KO tumor (from clone #345) stopped growing whereas all control tumors (from parental cells and control clones) still grew at a rapid path (Supplementary Figure S7C). Since gal-9-KO and WT MB49 cells had the same growth properties in vitro, our observations suggested that the progressive growth reduction of gal-9-KO tumors was related to a more efficient immune response in syngeneic mice. We suspected that the progressive reduction of tumor growth for gal-9-KO clones was related to some improvement in the anti-tumor immune response. To confirm this hypothesis, in parallel with the previously presented experiments, we performed serial tumor growth assays on athymic (nude) mice comparing the 3 gal-9-KO clones, the 2 WT-Ctrl clones and the parental MB49. As shown in Fig. 2C,D, in the context of athymic mice, tumors derived from the gal-9-KO clones did not undergo progressive growth reduction. On the contrary, they had a tendency to grow even faster than the WT cells at cycles 3 and 4 (individual growth curves are visible in Supplementary Figures S7D,E).

### T-cells are involved in the enhancement of the immune response resulting from gal-9 invalidation in MB49 cells

**Figure 3:**
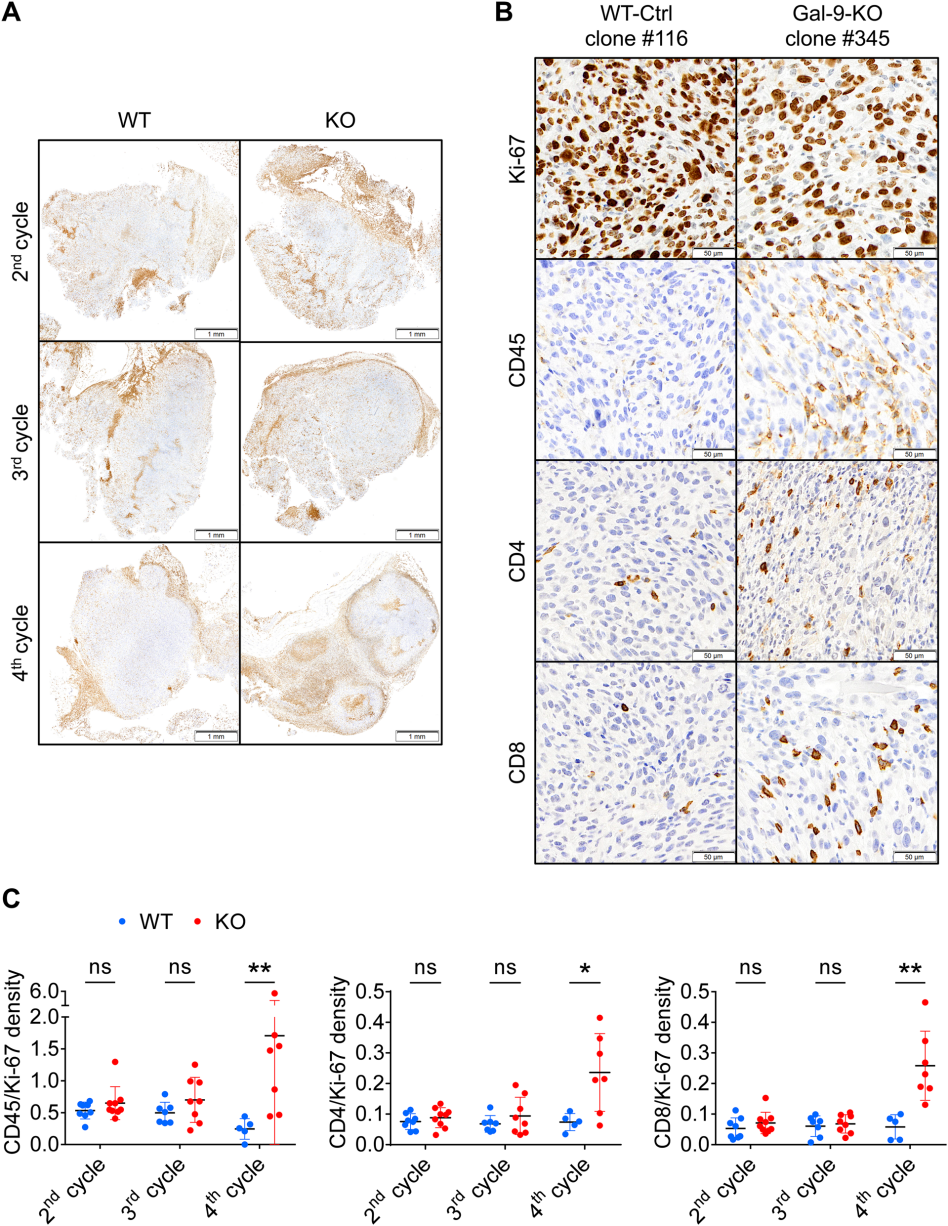
In gal-9-KO tumors, late stages of tumor growth reduction are associated with an increase in T-cells infiltrating tumor nodules. (**A**) Low magnification of CD45 staining on sections of WT and gal-9-KO tumors at cycles 2, 3 and 4 (tumors derived from clones # 116 and 345, respectively). At cycle 2, CD45 staining was detected almost exclusively in peri-nodular septa. However, for gal-9-KO tumors, at cycle 3 and even more at cycle 4, CD45 was becoming more and more abundant inside the tumor nodules. (**B**) High magnification of CD45, Ki-67, CD4 and CD8 staining on serial sections of a WT-Ctrl tumor (#116) and a gal-9-KO tumor (#345) at cycle 4. (**C**) Quantitative assessment of CD45, CD4 and CD8 staining on serial sections of tumors collected at cycles 2, 3 and 4 of tumor growth. Digitalization was performed in large Regions of Interest (ROIs) covering in as much as possible the whole tumor sections and excluding areas with absence or very low levels of Ki-67 staining, including peri-nodular septa. Therefore these ROIs were covering mainly the internal parts of the tumor nodules. Ratios of CD45, CD4 or CD8 to Ki-67 staining densities were acquired in these ROIs and plotted for each tumor growth cycles. Comparisons of mean ratios obtained for WT and KO tumors were subjected to Mann–Whitney tests for assessment of statistical significance.

**Figure 4:**
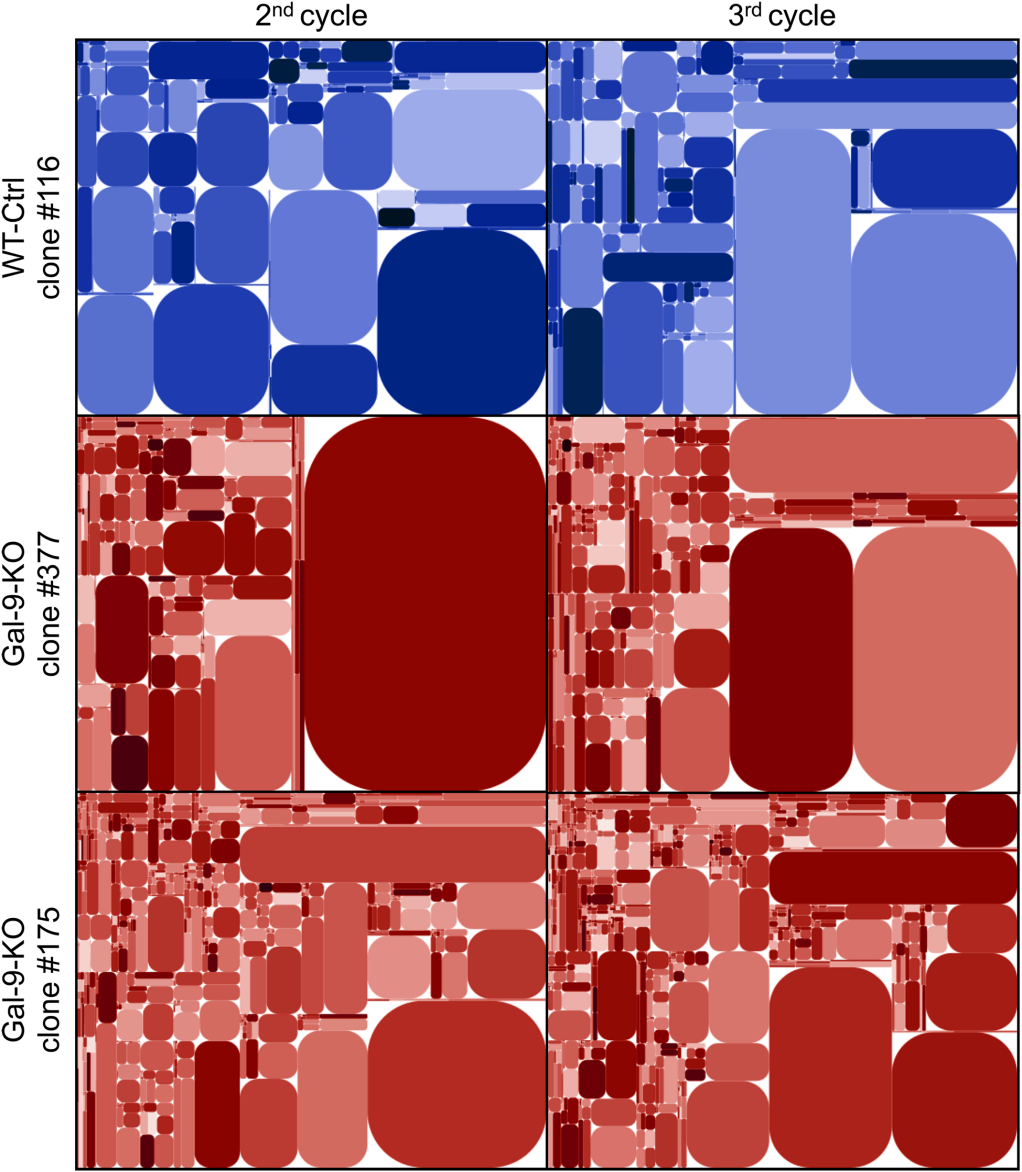
Modifications of T-cell clonotype profile at an early stage of tumor growth reduction in gal-9-KO tumors. The diversity of the CDR3 segment of the TCR β-chain was investigated using total tumor RNA subjected to reverse transcription, selective PCR amplification of the CDR3 coding region and deep sequencing. Here are shown tree maps of the clonotypes identified in one WT-Ctrl (#116) and two gal-9-KO (#175 and 377) tumors at the 2nd and 3rd cycles of tumor growth. These tree maps were generated as follows: the entire plot area was divided into sub-areas according to V-usage, then subdivided according to J-usage and again according to unique CDR3 sequences resulting from somatic hypermutations. Each clonotype is characterized by its unique sequence and the frequency of the reads matching this sequence. Those with a sufficient frequency of reads are represented by a rounded rectangle whose size is proportional to this frequency. At cycle 2, the number of clonotypes of average size (small rectangles) was greater in tumors derived from KO (#377 and #175) than from WT (#116) clones. This number was increasing from cycle 2 to cycle 3 but still with a greater abundance in tumors derived from gal-9-KO clones. Simultaneously, there was a relative size reduction of very large pre-existing clonotypes (represented by big rectangles). These tree map characteristics are in favor of a greater TCR diversity at cycle 3 compared to cycle 2 and in both cases for KO compared to WT tumors. For the precise numbers of unique sequences in the various experimental conditions, see Supplementary Table S1.

Because athymic mice are characterized by a complete absence of T-cell ontogenesis, these results suggested that the tumor growth reduction of the gal-9-KO clones was dependent on T-cells. To strengthen this hypothesis, we used immunohistochemistry to investigate T-cell infiltration of WT and gal-9-KO tumors grown on syngeneic mice. Tumor sections made at cycle 2, 3 and 4 were processed using antibodies reacting with Ki-67, CD45, CD4 and CD8. For each marker, density staining was quantified by digital imaging. Upon visual examination, we made two main observations. First, we noted that the bulk of leucocytes (CD45^+^), including T-cells (CD4^+^ and CD8^+^), were concentrated at the periphery of tumor nodules. Their abundance in these areas was identical for KO and WT tumors at all cycles of tumor growth (Fig. 3A). In contrast, leucocytes were quite rare inside the tumor nodules except for gal-9-KO tumors at cycle 4 for which a substantial number of CD45^+^, CD4^+^ and CD8^+^ leucocytes were visible inside the nodules (Fig. 3B). This was confirmed by digital assessment of the 4 markers (Ki-67, CD45^+^, CD4^+^ and CD8^+^) as shown by the histograms of Fig. 3C. The density of Ki-67 staining was used to normalize the density of the other markers. At cycles 2 and 3, densities of CD45, CD4 and CD8 staining inside the nodules were almost identical for KO and WT tumors. On the other hand, at cycle 4, staining densities of these 3 markers were much higher for KO than WT tumors. Overall, our conclusion was that T-cell infiltration of tumor nodules was specific for gal-9-KO tumors. However, this infiltration was detectable only at cycle 4, therefore at a late stage of the anti-tumor immune response, when tumor growth was already strongly impaired (5/8 KO tumors were of minimal size, weighing less than 70 mg). These observations suggested that, upstream of T-cells infiltration, some immune-related events were occurring in a different manner in gal-9-KO and WT tumors, at an earlier stage of the serial transplantation assay, possibly during cycles 2 or 3. A glimpse of these early events was given by quantitative assessment of the diversity of the stromal T-cell repertoire. Using total tumor c-DNA, the diversity of the TCR complementary determining region 3 (CDR3) was assessed by PCR amplification and high throughput sequencing (Fig. 4). This analysis resulted in clonotypes characterized by a unique sequence and a frequency of reads matching this sequence. Figure 4 and Supplementary Table S1 show a greater number of clonotypes in KO by comparison with WT tumors and at cycle 3 by comparison with cycle 2. In gal-9-KO tumors, a substantial number of emerging clonotypes were of average size (between 100 and 1000 reads) therefore likely to be representative of functional T-cell clones. In summary, these observations suggested that the T-cell repertoire had a tendency to increase its diversity from cycle 2 to cycle 3 while being, in both cases, more diverse in KO than in WT tumors. In other words, it was suggested that long before intra-nodular T-cell infiltration, as early as the second cycle of tumor growth, changes in T-cell distribution and behavior were taking place preferentially in gal-9-KO tumors.

### Gal-9 ablation enhances transcription of genes related to interferon-γ response

**Figure 5:**
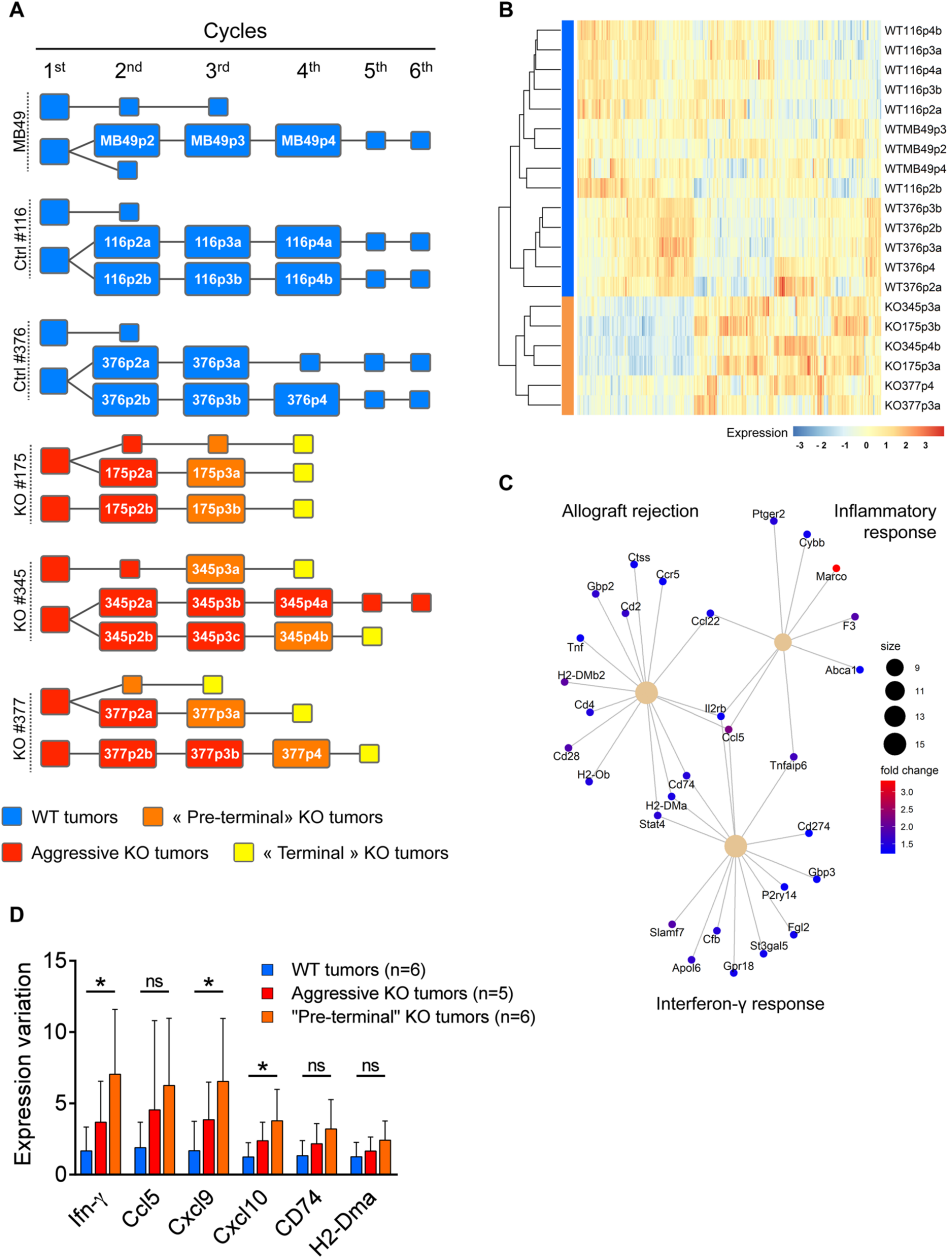
RNAseq analysis of WT and gal-9-KO tumors at different cycles of in vivo tumor growth. (**A**) Cartoon depicting the serial tumor transplantation assay with emphasis on the tumor “lineages” and their changes in growth and aggressiveness : WT tumors are represented in blue, “aggressive” (WT-like) KO tumors in red, “terminal” (infiltrated by T-cells and exhausted) KO tumors in yellow and the “pre-terminal” KO tumors in orange. The annotated rectangles represent tumors used for RNA and/or protein analyses. Other tumors are represented by squares without annotations. The complete set of investigations performed on each tumor of this assay is shown in Supplementary Figure S6. (**B**) Heatmap representing the expression profile of the 344 most differentially expressed genes between WT and “pre-terminal” KO tumors, based on RUVseq (k = 3) and DEseq2 analysis (fold change > 2, adjusted *p* value < 0.05). (**C**) Enrichment analysis of the differentially expressed genes within the “hallmark” gene sets defined in the clusterProfiler package (Bioconductor): 15 up-regulated genes in the “pre-terminal” KO tumors are associated with the “allograft rejection”, 9 with the “inflammatory response” and 15 with the “interferon-γ response”. (**D**) RT-qPCR for quantitation of mRNAs transcribed from the *interferon-γ* gene and 5 genes whose expression is known to be often stimulated by interferon-γ (encoding the CCL5, CXCL9 and CXCL10 chemokines and the MHC class II components CD74 and H2-DMA). Comparative assessment was made in WT (n = 6), “aggressive” KO (n = 5) and “pre-terminal” KO tumors (n = 6). Interferon-γ, CXCL9 and CXCL10 m-RNAs are significantly more abundant in “pre-terminal” gal-9-KO than in WT tumors (Kruskal–Wallis test followed by Dunn’s multiple comparisons test).

To further investigate biological events occurring prior to T-cell infiltration and blockade of tumor growth, we performed a comparative transcriptome analysis of gal-9-KO and WT tumors through the successive cycles of the serial tumor transplantation assay (Fig. 5A). Our aim was to find correlations between the dynamics of transcriptional changes and the progressive inhibition of gal-9-KO tumor growth. RNAseq data from 30 samples were subjected to various types of comparative bioinformatics analyses. Unsupervised clustering as well as supervised comparisons did not achieve separation of gal-9-KO from WT tumors or early from late passages while there was a trend towards clusterization of tumors derived from the same MB49 clones (Supplementary Figure S8). It seems that biological characteristics related to the progressive immune response were, to a large extent, relegated to the background by other clonal private characteristics. We reasoned that it was not sufficient to compare the whole set of WT tumors with the whole set of KO tumors. We thought that it was possible to gain sensitivity by focusing the comparison on the subset of KO-tumors taken at the late stages of the serial transplantations, especially at the last cycles preceding the collapse or quasi-collapse of tumor growth, hereafter named “pre-terminal cycles” (Fig. 5A). Our bet was that it would be easier to find transcriptional characteristics consistent with the enhancement of the immune response in this subset of “pre-terminal” tumors. Reciprocally, these characteristics were expected to be absent or less visible in the rest of the KO tumors, non-pre-terminal tumors thereafter called “aggressive” KO tumors (Fig. 5A). Combining the RUVseq package^25^ with the DESeq2 software we generated a list of differentially expressed (DE) genes resulting from the comparison of “pre-terminal” KO vs all WT tumors. This list of 344 genes resulted in effective separation of “pre-terminal” gal-9-KO from WT tumors as shown by the heat map in Fig. 5B. This same list was subjected to enrichment analysis using the package ClusterProfiler^26^. The greater enrichment scores were obtained for the following general functions: cell response to interferon-γ, allograft rejection and inflammatory response (Fig. 5C). One gene related to these 3 general functions—*CCL5* (also called *Rantes*)—appeared to be strongly expressed in “pre-terminal” gal-9-KO tumors. Seeking confirmation of the enhancement of interferon-γ response in “pre-terminal” KO tumors, we investigated by q-RT-PCR the abundance of interferon-γ m-RNA along with mRNAs encoding 3 chemokines (CCL5, CXCL9 and CXCL10) and two components of the MHC II complex (CD274 and the α-chain). The expression of these transcripts is known to be often enhanced by interferon-γ. For each of them, we found a greater abundance in pre-terminal KO tumors than in WT tumors. The differences were statistically significant for interferon-γ, CXCL9 and CXCL10 (Fig. 5D).

### Progressive increase in the concentrations of CXCL10 in gal-9-KO tumors

**Figure 6:**
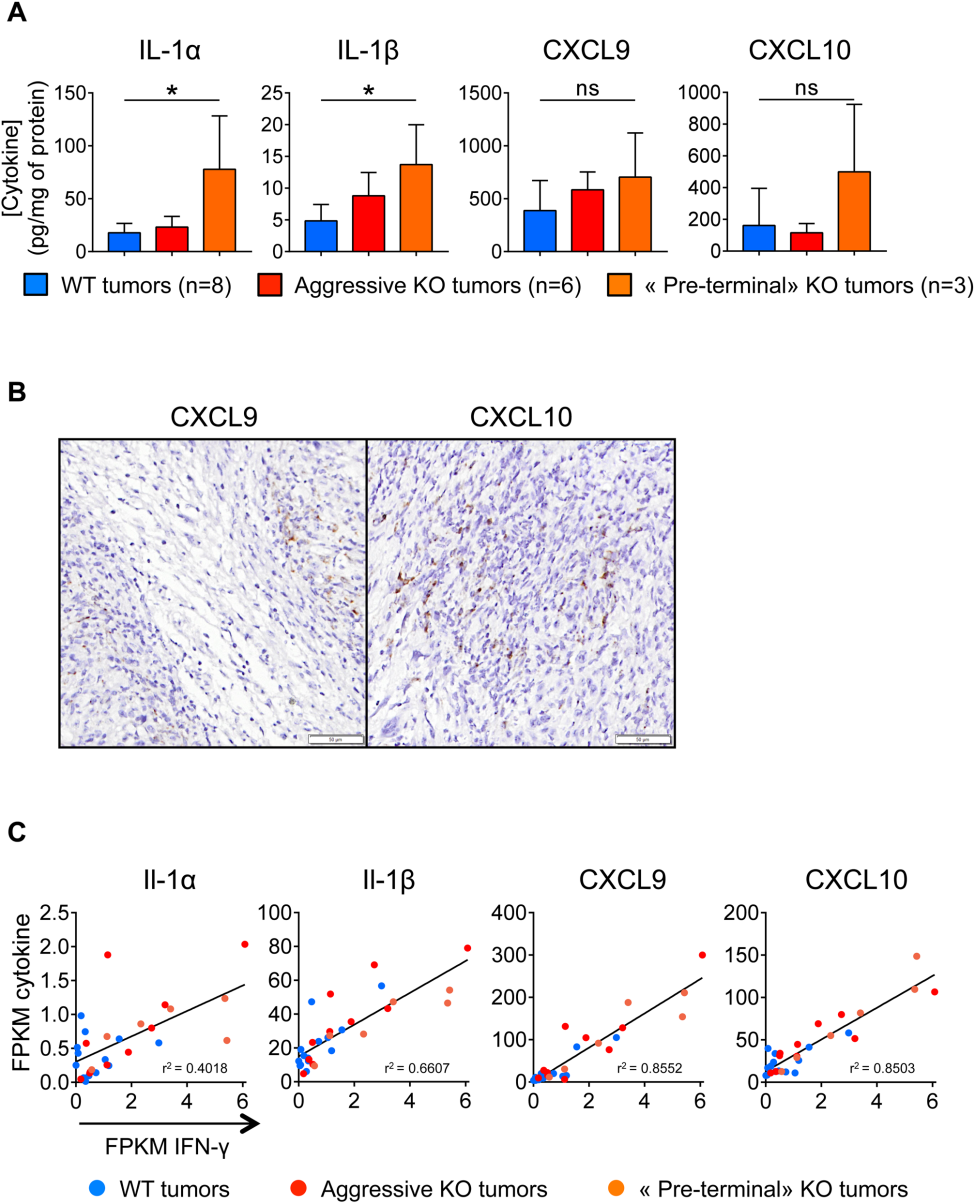
Cytokine status in gal-9-KO and WT tumors. (**A**) Concentrations of four cytokines resulting from a multiplex ELISA performed on 31 cytokines detected in tumor protein extracts at various cycles of tumor growth (8 WT tumors, 6 aggressive KO tumors and 3 “pre-terminal” KO tumors). For Il-1 α and β, there was a significant increase in their concentrations in the extracts of “pre-terminal” KO tumors compared to the WT. CXCL9 and CXCL10 concentrations were also greater in “pre-terminal” KO tumors, although the differences were not statistically significant. However, these observations are worth to mention because the concentrations of CXCL9 and CXCL10 in tumor extracts were greater than those of other cytokines by 2 orders of magnitude even at baseline (WT tumors). (**B**) RNAscope detection of CXCL9 and CXCL10 transcripts in “pre-terminal” KO tumors. Signals related to CXCL9 and CXCL10 mRNAs are mainly detected in malignant epithelial cells which often have a basal-like appearance, often located at the periphery of tumor nodules (this is best illustrated for CXCL9 in the left panel). (**C**) Abundance of the messenger RNAs of the above-mentioned cytokines (FPKM values from RNAseq data) as a function of the amount of IFN-γ transcripts. For the four cytokines mentioned in (**A**), there is a strong positive linear correlation between their level of expression and that of interferon-γ. This correlation is observed in KO as well as in WT tumors. However, tumors combining highest expressions of interferon-γ combined with highest expressions of IL-1 α or IL-1 β, CXCL9 or CXCL10 were “pre-terminal” tumors.

In order to better understand the immune mechanisms of the progressive growth inhibition of gal-9-KO tumors, we used a multiplex ELISA assay to investigate the expression of a panel of 31 cytokines in the tumor cells and their microenvironment. This assay was carried on whole proteins extracted from WT and KO tumor fragments collected at cycle 2 and 3 of tumor growth. Cytokine concentrations were compared between these 3 sets of tumors: WT, aggressive gal-9-KO and “pre-terminal” gal-9-KO tumors (Fig. 6A). Significant differences between “pre-terminal” KO tumors and WT tumors were found for 2 cytokines—IL-1 α and β—which were more abundant in “pre-terminal” KO tumors than in WT tumors. The concentrations of CXCL9 and CXCL10 were also increased in “pre-terminal” KO tumors, although the differences with WT tumor did not reach statistical significance. Nevertheless, we retain this observation for our subsequent investigations for two reasons. First, as shown in Fig. 5D, CXCL9 and CXCL10 mRNAs were more abundant in pre-terminal than in WT tumors with statistical significance. Next, we were struck by the facts that, even at baseline (WT tumors), the concentrations of CXCL9 and CXCL10 in tumor extracts were greater than those of other cytokines by one or two orders of magnitude. This suggested that these two cytokines were produced, at least to a large extent, by malignant cells. To confirm this assumption we performed detection of their transcripts on tissue sections using the RNAscope technology. As shown in Fig. 6B, signals related to CXCL9 and CXCL10 mRNAs were mainly observed in malignant epithelial cells. In an attempt to connect results based on protein detection and RNAscope with the data resulting from transcriptome profiling, we investigated a potential correlation between the abundance of the transcripts encoding the four cytokines overexpressed in “pre-terminal” KO tumors and the mRNAs encoding interferon-γ. As shown in Fig. 6C, for these four cytokines, there was a strong positive linear correlation of their expression with interferon-γ expression in KO and WT tumors. The highest levels of concomitant expression for interferon-γ on the one hand and IL-1 α or β, CXCL-9 or 10 on the other hand, were recorded for “pre-terminal” tumors. This was one additional observation suggesting a critical role of interferon-γ in the progressive deployment of the anti-tumor immune response.

### Gal-9 gene ablation in MB49 cells enhances in vitro CXCL10 induction in response to recombinant interferon-γ

**Figure 7:**
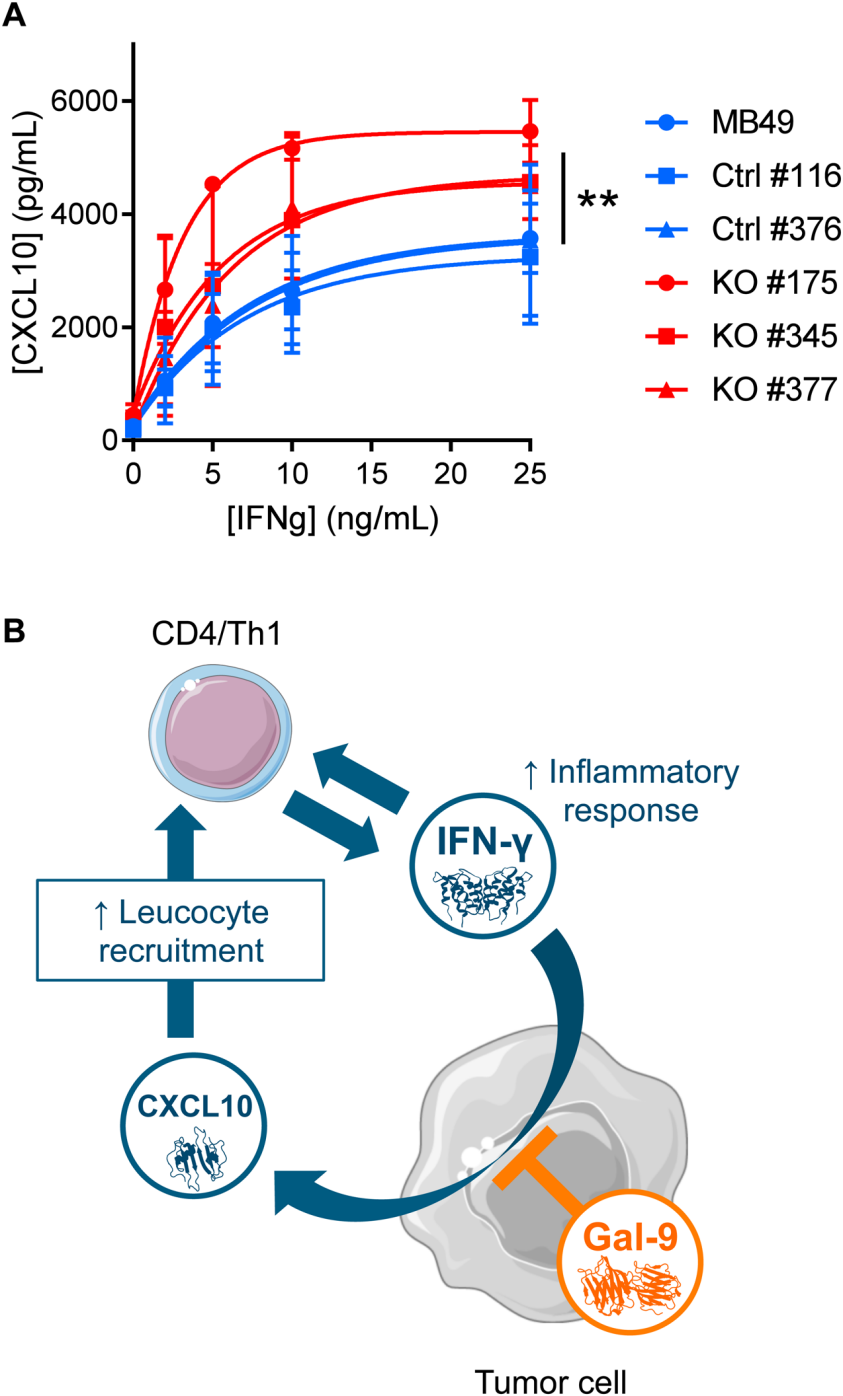
Gal-9 expressed by malignant epithelial cells inhibits CXCL10 induction resulting from stimulation by interferon-γ (**A**) Comparative assessment of CXCL10 production by gal-9-KO and WT MB49 cells stimulated in vitro by recombinant interferon-γ. Conditioned media were collected after 72 h of stimulation with increasing concentrations of interferon-γ and subjected to CXCL10 ELISA assay. At 25 ng/mL of interferon-γ there was a significant difference of CXCL10 concentration in the medium between KO [KO#175 (n = 2); KO#345 (n = 2); KO#377 (n = 2)] and WT cells [MB49 (n = 2); Ctrl#116 (n = 2); Ctrl#376 (n = 2)](Mann–Whitney test). (**B**) Proposed model of gal-9 attenuation of the amplification loop involving interferon-γ and CXCL10. We assume that in the tumor microenvironment in vivo, interferon-γ released by activated T-cells stimulates the production of CXCL10 by MB49 cells. In turn, CXCL10 stimulates T-cell infiltration, therefore boosting the anti-tumor immune response. However, the expression of gal-9 by malignant cells reduces their sensitivity to interferon-γ therefore slowing down the amplification loop finally making the immune response weaker. So far, we do not know whether this inhibition is due to intra-cellular gal-9, gal-9 bound to the cell-surface and/or gal-9 released in the extra-cellular medium. Therefore, in our cartoon, the symbol of gal-9 is positioned with partial overlap of the intra-cellular structures, plasma membrane and extracellular medium.

In order to confirm the hypothesis of a greater sensitivity of MB49 cells to their stimulation by interferon-γ in the absence of gal-9, we performed the following experiment in vitro. Gal-9-KO and WT cells were subjected to a 72 h treatment with increasing concentrations of recombinant interferon-γ (from 2 to 25 ng/ml). Then, the concentration of CXCL10 was assayed in the conditioned medium. As shown in Fig. 7A, CXCL10 production was completely absent at baseline. It was induced in a dose-dependent manner by interferon-γ. This induction was much stronger for gal-9-KO clones than for WT clones or MB49 parental cells. This was a confirmation of the greater sensitivity of MB49 cells to their stimulation by interferon-γ in the absence of gal-9.

## Discussion

The aim of this study was to build a murine tumor model designed to investigate the role of gal-9 in host-tumor relationships in the context of immunocompetent syngeneic animals. Our approach was based on the production of MB49 isogenic clones with or without gal-9 expression. These clones were generated by the CRISPR/Cas9 method of gene editing. One major advantage of this approach was to guaranty that the silencing of the gal-9 gene would be irreversible. One disadvantage was the possibility of off-target genetic lesions that could randomly affect the phenotype of edited clones in a way which had nothing to do with gal-9 biology. Another source of heterogeneity was the cloning step taking place after transfection of the editing material. In order to minimize the influence of unwanted clonal heterogeneity, we have compared the gal-9-KO clones with control clones subjected to the same editing process but having retained gal-9 expression. They were used in addition to the parental MB49 cells through the whole study. No differences in terms of tumor growth were apparent when gal-9-KO or WT-Ctrl cells were injected subcutaneously. However serial transplantations of tumor fragments unmasked a completely different pattern of growth for gal-9-KO and Ctrl-cells. Gal-9-KO tumors were affected by a progressive inhibition of tumor growth resulting in minute, “exhausted” tumors at the third or fourth cycles of growth. This reduction of tumor growth was dependent on the immune response and involved T-cells since it was not observed when the serial transplantations were made into nude mice.

Our investigations based on IHC, RNAseq and cytokine profiling have confirmed the progressive enhancement of the immune response through 2 or 3 cycles of tumor growth. T-cell infiltration in the tumor nodules was only detected in terminal “exhausted” tumors. However, this change of histological pattern concomitant of growth termination was preceded by transcriptional changes in the micro-environment of “pre-terminal” tumors i.e. tumors taken at the last cycle of growth prior to the exhaustion. A fraction of these changes was highly suggestive of the emergence of an immune response. Enrichment analysis of genes differentially expressed between pre-terminal KO and WT tumors have highlighted three main processes related to the immune response: cell response to interferon-γ, allograft rejection and inflammatory response. The *CCL5* mRNA was standing at the intersection of the three sets of genes highlighted by the enrichment analysis. This is consistent with a recent publication reporting the crucial role of CCL5 produced by malignant cells to stimulate the penetration of tumor infiltrating lymphocytes in human malignancies and murine tumor models^27^.

Profiling of the T-cell repertoire by deep sequencing has unveiled critical events in host-tumor interactions which were taking place in KO-tumors upstream of pre-terminal tumors as early as the second cycle of tumor growth. Indeed, already at this stage, the repertoire of T-cell clonotypes was more diverse for gal-9-KO tumors. Thanks to our data obtained by IHC, we know that, at this stage, the overwhelming majority of T-cells are contained in perinodular margins. Therefore our observations on the T-cell repertoire suggest a mobilization of T-cells in these margins taking place before nodular invasion.

Overall, our data show that for tumors derived from gal-9-KO clones, the deployment of the immune response leading to tumor growth arrest requires 2 simultaneous conditions: (1) absence of gal-9 produced by tumor cells; (2) serial transplantation for 2 or 3 cycles of tumor growth. We now have to discuss by which mechanisms these two conditions are suspected to contribute to the anti-tumor immune response.

The contribution of gal-9 ablation to the enhancement of the immune response against MB49 tumors is consistent with several inhibitory effects of extra-cellular gal-9 on immune effectors cells which have been well documented in the murine context. These effects are mainly death of CD4^+^ Th1 cells, expansion of MDSCs and functional impairment of natural killer cells^4,11,12^. Independently of these cell non-autonomous mechanisms, our investigations based on serial MB49 tumor transplantations has pointed to potential mechanisms of immune escape intrinsic to malignant cells. First, transcriptional studies have suggested a better response to interferon-γ in tumors resulting from gal-9-KO cells. This was confirmed by additional investigations focused on cytokines. Cytokine profiling on tumor protein extracts showed a substantial increase in the expression of IL-1α and β, CXCL9 and CXCL10 in pre-terminal gal-9-KO tumors by comparison with WT and aggressive gal-9-KO tumors. In addition, going back to the transcriptional level, we found remarkable correlations between the abundance of interferon-γ transcripts and those of the above-mentioned cytokines including CXCL9 and CXCL10 which were shown to be produced by malignant cells (Fig. 6B,C). Finally, we obtained direct evidence of the influence of gal-9 expression on tumor cell response to interferon-γ by in vitro comparative stimulation of gal-9-KO and WT MB49 clones. All these cells responded to recombinant interferon-γ by a de novo, dose-dependent release of CXCL10. However, this release was much more abundant for gal-9-KO clones than for WT-Ctrl clones or parental cells. This suggests that gal-9 impairs MB49 cell response to interferon-γ. So far, we do not know whether it exerts this effect when it is trafficking inside the cells or when it is bound to their surface and/or released in the extra-cellular medium. Although the mechanism of this alteration is not known, we can speculate that gal-9 slows down a feed-forward loop involving CD4^+^ Th1 + T-cells, interferon-γ, malignant cells and CXCL10 as explained in the cartoon on Fig. 7B. Our data showing that gal-9 produced by MB49 cells impairs the anti-tumor immune response probably by both cell-autonomous and cell-intrinsic mechanisms seems to contradict reports of a better prognosis for a category of human malignancies—especially breast carcinomas—with abundant expression of gal-9^18^. To cope with this paradox, one has to keep in mind that some biomolecules can have both pro- and anti-tumor effects. Intra-cellular gal-9 is suspected to decrease the metastatic potential of malignant cells but this might be achieved at the cost of some immunosuppressive effects^18^. In the future, concomitant detections of interferon-γ and CXCL10 in tumor tissue sections and maybe in plasma samples might help to assess the immunosuppressive effects of gal-9 in various tumor contexts.

Serial transplantations are common in experimental hematology and onco-hematology, especially in studies dealing with stem cells or tumor initiating cells^28,29^. In contrast, to our knowledge, this method has not been previously reported for solid tumors grafted in syngeneic mice. It was introduced in our experimental system partly by chance while we were seeking better conditions of tumor growth which were achieved by transplantation of tumor fragments instead of cells expanded in vitro. Then, we realized that serial transplantations were required to unmask the greater vulnerability of gal-9-KO tumors to the immune response. On the side of malignant cells, it is difficult to consider a type of immune-editing that would result in the selection of cells with greater sensitivity to the immune response. In fact, it would be the contradiction of the concept of immuno-editing. On the other hand, tumor fragments used for serial transplantation are the only possible “vectors” going from one mouse to another with the capacity to increase the efficiency of the immune response. Since transplanted tumor fragments contain infiltrating leucocytes, their implantation in the recipient mice may result in the adoptive transfer of immune cells which may facilitate and accelerate the onset of the immune response in the recipient mice. This effect might be enhanced by concomitant mechanical factors: (1) the mincing of small tumor fragments may increase their immunogenicity; (2) the sub-cutaneous insertion of these fragments undergoing partial necrosis is likely to increase local inflammation in the tumor niche. Investigating the role of immune cells contained in tumor fragments and passively transferred from one mouse to another is conceivable but would require a dedicated project. However, on the basis of our data, we can already consider serial transplantation in syngeneic mice as a possible approach to refine the exploration of some mechanisms of tumor immune tolerance or rejection. It seems that it might help to investigate the maturation of host-tumor relationships through a greater period of time than what is possible when working with transplantation in a single mouse. Indeed, except for tumor models growing very slowly, it is usually difficult to extend for more than 3 to 4 weeks the investigations made on tumors grafted on a single animal, without breaking practical and/or ethical limits. Therefore, serial transplantation might prove useful to better mimic the evolution of host-tumor relationships as it takes place in the patients where tumor history usually spans several months or even several years.

## Material and methods

### MB49 cell line

MB49 is a murine bladder carcinoma cell line derived from the C57BL/6 mouse strain which was purchased from Merck Inc (Millipore Cat# SCC148, RRID:CVCL_7076). Cells were grown in DMEM medium (Gibco, Life Technologies) supplemented with 5% fetal calf serum, 2 mM glutamine and 5 µg/mL gentamycin.

### Ablation of the gal-9 gene in the MB49 cells

Our strategy to invalidate the gal-9 gene was to make a deletion framing the start codon by concomitant transfection of 2 guide RNAs. The sequences of these guide RNAs were designed in silico using the CRISPOR tools^30^. They were matching a sequence in the first exon and first intron of the gal-9 gene, respectively. The DNA inserts encoding the upstream and downstream guide RNAs were generated from two pairs of single strand oligonucleotides, respectively (F: 5′-CACCGCTGTCGTCCACCATCGAGTG-3′; R: 5′-AAACCACTCGATGGTGGACGACAGC-3′) and (F: 5′-CACCGAGTCGCCGTGTGTGCAGGT-3′; R: 5′-AAACACCTGCACACACGGCGACTC-3′). These two pairs of oligonucleotides were annealed by denaturation and slow renaturation prior to vector insertion. These inserts were ligated to a backbone Lenti-CRISPR-V2puro plasmid (RRID:Addgene_52961) to co-express the guide RNA sequences with *Streptococcus pyogenes* Cas9 linked either to the GFP or mCherry sequence by a P2A sequence encoding a self-cleaving peptide. These plasmids were digested by *BsmBI* and ligated with the annealed guide RNA sequences. Cell lines were transfected with both GFP and mCherry plasmids, using the TurboFect (Thermo Scientific) reagent according to the manufacturer’s instructions. They were harvested, 48 h after transfection, sorted according to their double GFP/mCherry positive expression and automatically seeded at one cell/well in a 96 well cloning plate (BD Influx). After 2 weeks, emerging clones were tested for their gal-9 expression by intracellular staining using rat anti-mouse gal-9 antibody clone 108A2 conjugated with allophycocyanin (APC) (BioLegend Cat# 137,911, RRID:AB_2750154); they were analysed with an Accuri C6 flow cytometer (BD Biosciences). After sequencing analysis confirming their knock-out status, 3 of them were selected for the study (KO # 175, 345 and 377). In addition, we selected 2 clones having undergone the same transfection treatment, sorting and clonal selection, but having preserved an intact gene sequence at the sites targeted by the guides and still strongly expressing gal-9 (Ctrl # 116 and 376). Furthermore, we generated a list of the most probable off-targets by in silico analysis (Supplementary Table S2 and Supplementary Figure S4) and compared the FPKM values of these transcripts in KO clones with the controls and parental lines. No differences went outside this analysis. Exome sequencing has been performed for authentication of MB49 parental cells and derived clones (3 gal-9-KO and 2 control clones). The corresponding data are publicly available on the ArrayExpress database of the European Bioinformatics Institute (http://www.ebi.ac.uk/arrayexpress) with the following accession number: MTAB-9836.

### Genomic DNA extraction, PCR and sequencing analysis

Cells were lysed in Tri-reagent (MRC) and genomic DNA isolated according to the manufacturer’s instructions, using chloroform for the phase separation. DNA quality was assessed by spectrophotometry (Thermo Scientific Nanodrop 2000 microvolume spectrophotometer, RRID:SCR_018042). PCR tests were performed (F: 5′-CCGTGCTAGATCAGGCTCTG-3′; R: 5′-TGGCCATGTGACTGGTTCTC-3′) to amplify the region targeted by the guide RNAs, with a high-fidelity DNA polymerase Q5 (New England BioLabs) (amplicon size = 1360 bp for the WT). After migration on a 1.5% agarose gel, PCR products were extracted with DNA/RNA extraction/purification kit (SmartPure Gel kit, Eurogentec). After quality assessment, samples were sequenced by Sanger sequencing (Eurofins) using both forward and reverse primers. Sequencing results were analysed using FinchTV software.

### Protein extraction and western blotting

Experiments were performed as previously described in Lhuillier et al.^31^. The following primary antibodies were used: polyclonal goat anti-mouse gal-1 (R&D Systems Cat# AF1245, RRID:AB_354693), mouse anti-human/murine gal-3 clone B2C10 (Santa Cruz Biotechnology Cat# sc-32790, RRID:AB_627657), polyclonal rabbit anti-human/murine gal-8 (#MBS828618, MyBioSource), rat anti-mouse galectin-9 clone 108A2 (BioLegend Cat# 137,901, RRID:AB_10568691); mouse anti-β-actin clone AC-74 (Sigma-Aldrich Cat# A2228, RRID:AB_476697).

### Cell proliferation assay

MB49 cells (WT and KO) were seeded in a 96-well plate at the rate of 2000 cells/well. The proliferation rate in 24 h was determined by measuring the ATP concentration in the wells at 24 and 48 h (ATPlite Luminescence Assay System, PerkinElmer).

### RNAseq analyses

RNAseq analyses were performed in 2 distinct stages for in vitro cultured cells and tumor cells transplanted on syngeneic mice.

In vitro cultured cells were lysed in Tri-reagent (MRC) and total RNA isolated according to the manufacturer’s instructions, using chloroform for the phase separation. RNA quality was assessed by spectrophotometry (Thermo Scientific Nanodrop 2000 microvolume spectrophotometer, RRID:SCR_018042). RNA sequencing and analysis were performed by IntegraGen (Evry, France). The raw data (bam files) are available on ArrayExpress under the following accession number: E-MTAB-9570. The quality of reads was assessed for each sample using FastQC. Fastq files were aligned to the reference Mouse genome mm10 with STAR (STAR, RRID:SCR_015899). Reads mapping to multiple locations were removed. Gene expression was quantified using the full Gencode vM18 annotation. STAR was also used to obtain the number of reads associated to each gene in the Gencode (GENCODE, RRID:SCR_014966) vM18 database (restricted to protein-coding genes, antisense and lincRNAs). Bioconductor DESeq package was used to generate the count matrix. After normalizing for library size, the count matrix was normalized by the coding length of genes to compute FPKM scores (number of fragments per kilobase of exon model and millions of mapped reads) (internal IntegraGen document and Anders et al. and Wang et al.^32,33^).

When dealing with tumors carried by syngeneic mice, RNA was extracted from frozen tumor fragments (30 mg) using the RNAeasy kit (Qiagen) according to the manufacturer’s instructions. During the cell lysis step, tumor fragments were crushed with TissueRuptor (Qiagen) in RLT buffer supplemented with 40 mM dithiothreitol (DTT). RNA concentration and quality were assessed with a Bioanalyzer instrument (Agilent). RNA sequencing and analysis were performed by Novogene (Cambridge, UK). The raw data (bam files) are available on ArrayExpress under the following accession number: E-MTAB-9220. Sequencing libraries were generated using the NEBNext Ultra RNA Library Prep Kit for Illumina (NEB, USA). Index-coding was done by ligation of sequencing adaptors. Following PCR enrichment, index-coded samples were clustered using a cBot Cluster Generation System with the PE Cluster Kit cBot-HS (Illumina). Then libraries were sequenced on an Illumina platform and paired-end reads were generated. Raw data of FASTQ format were first filtered to remove reads with adaptor contamination and reads with low quality. The Q20, Q30 and GC content of the clean data were calculated. Downstream analyses were based on the paired-end clean reads which were aligned to the reference genome using the STAR software (*Mus musculus* GRCm38). The HTSeq software (HTSeq, RRID:SCR_005514) was used to count the read numbers mapped of each gene. Then FPKM (Fragments Per Kilobase of transcript sequence per Million base pairs sequence) was calculated for each gene; it takes in account gene lengths in addition to mapped reads counts.

Differential expression was performed on raw counts of RNAseq, by applying the DESeq2^25^ package, version 1.26.0, combined with the RUVseq (RUVSeq, RRID:SCR_006263) package^34^, version 1.20.0 (both in R language). We identified genes with absolute value of fold change > 2 and FDR adjusted p value of F test < 0.05 as differentially expressed (DE). Heatmaps of DE genes were generated with Pheatmap (pheatmap, RRID:SCR_016418) R package, version 1.0.12, after a transformation of raw counts using the function of variance stabilizing transformation of DESeq2.

Gene ontology was explored using the Bioconductor ClusterProfiler package^26^ version 3.14.3, with DE genes identified from DESeq2 analysis. Over-representation and gene set enrichment analysis were done against MSigDb (Molecular Signatures Database, RRID:SCR_016863) gene sets, which were loaded through the MSigDb package version 7.1.1, and genome wide annotation for Mouse, sourced from org.Mm.eg.db package version 3.10.0 (available at www.bioconductor.org). Enrichment analyses were later visualized by enrichplot (R package, version 1.6.1) (www.bioconductor.org).

### M-RNA quantification by RT-qPCR

Following total RNA extraction, reverse transcription was made using the “High capacity cDNA reverse transcription kit” (#4368814, LifeTechnologies), q-PCR was done using TaqMan Real-Time PCR assays from Thermo Scientific according to the manufacturer instructions. Four house-keeping genes were used for normalization (*GUSB*, *GAPDH*, *PPIA*, *RPLP0*).

### Animal care and tumor growth assays

Animal procedures were performed according to protocols approved by the Ethics Committee for animal experimentation n°26 certified by the French ministry of agriculture (“Comité d’Ethique en Expérimentation Animale n°26”) (project number n°20171108124492). Female C57BL/6 N were purchased from Janvier Labs (France) and housed in the Gustave Roussy animal facility. Mice were used between 8 and 12 weeks of age. Overall, 200 mice were used for this study. Animal care was done as previously described^35^: mice were housed in pathogen-free conditions in filter cap cages holding a maximum of 5 animals with irradiated aspen chip bedding and cotton fiber nesting material. They were maintained on a 12/12 light/dark cycle, with ad libitum UV-treated water and RM1 rodent diet. The animals were monitored every day for signs of pain, such as immobility or restlessness, reduction of drinking and food intake. The persistence of abnormal behaviors for more than one day led to the euthanasia of animals with suffering presumption. Prior to tumor collection, mice were sacrificed by cervical elongation. Otherwise, mice were euthanatized by carbon dioxide asphyxiation. Body weight and tumor volume of all mice were measured at least twice a week. In any event, the animals were euthanized, when their tumor size had reached the limit of 1.7 cm^3^. Before and during tumor transplantations, mice were anesthetized with a 2L/min continuous flow of 2% isofluorane/oxygen mixture, using a rodent anesthesia work station (Anesteo, France). For initial tumor inoculation, in vitro expanded cells were injected subcutaneously (5.10^5^ cells suspended in 100 μL of PBS). After 10 to 20 days of sub-cutaneous growth and mouse euthanasia, tumors were collected, weighted and cut in small fragments (70 or 100 mg) which were then subcutaneously engrafted on recipient mice. Tumor dimensions were measured every day using a digital caliper, starting from day 4 or 5 post-graft, and volumes were calculated based on the formula: width^2^ × length/2. In addition, tumors collected at the time of sacrifice were weighed. A part of them was fixed in 4% paraformaldehyde in order to carry out IHC and/or RNAscope analysis, and the rest was frozen for RNA/protein extractions. Overall, our animal studies were conducted according to the ARRIVE guidelines (Animal Research: Reporting of In Vivo Experiments). Particularly, physical randomization of recipient mice based on ear tag numbers was done prior to tumor transplantation. Partial blinding was applied to outcome assessment: tumor measurements were made in a random order by a person not aware of the tumor status (gal-9-KO or wild-type). Statistical methods are described in a later part of the Material and methods section and in the figure legends.

Statement: As the corresponding author, on behalf of all authors, Dr Pierre Busson certifies that all experiments performed on mice were done according to the relevant guidelines and regulations of the French ministry of agriculture with the assistance and under the supervision of the Gustave Roussy committee for animal welfare.

### Histology and immunohistochemistry

After fixation of tumors in 4% PFA, paraffin-embedded tumors were cut into 4 µm thick sections. At least one section per tumor was stained with hematoxylin–eosin-saffranin (HES) for quality control. For Ki-67 and CD8 detection, slides were incubated with rabbit monoclonal antibodies specific of Ki-67 (1:500) (Cell Signaling Technology Cat#  12202, RRID:AB_2620142) and CD8 (1:200) (Cell Signaling Technology Cat#  98941, RRID:AB_2756376). Primary antibody binding was revealed with the Rabbit PowerVision Kit (UltraVision Technologies) and the DAB PowerVision kit (ImmunoVisionTechnologies Co.). For detection of CD45 and CD4, slides were incubated with rat monoclonal antibodies specific of CD45 (#10051.05, 1:100, Histopathology) and CD4 (1:100) (Thermo Scientific Cat# MA1-146, RRID:AB_2536856). Primary antibody binding was revealed with Polink 2 plus rat HRP kit (GBI Labs). All antibody applications were performed with a Bond RX automated Leica Slide Stainer. Image acquisition was performed with a Virtual Slide microscope VS120-SL (Olympus, Tokyo, Japan), 20X air objective (0.75 NA, 345 nm/pixel). Quantification of immunostaining was done using the Definiens Tissue Studio software (Definiens AG, Munich, Germany). For all markers, staining densities were expressed as a number of stained cells—equivalent to stained nuclei—per mm^2^ of analyzed tissue. For Ki-67 which is concentrated in the nuclei, we used an algorithm performing automatic detection of nuclei stained with DAB according to their spectral properties. Positive and negative nuclei were counted in a manually selected Region of Interest (ROI) (excluding areas of necrosis, preparation artifacts, etc.). For CD45^+^, CD4^+^ and CD8^+^ staining, positive cells were detected using another algorithm performing detection of stained “objects” with a high probability of being single cells.

### In situ hybridization of mRNAs in tissue sections

The detection of mRNAs in tumor sections was made by in situ hybridization using the RNAscope technology (ACD and Biotechne). Fixed (4% PFA) and paraffin-embedded tumors were cut into 5 + /− 1 µm sections using a microtome. The paraffin ribbons were mounted on Superfrost Plus slides and deparaffinized before starting the RNAscope workflow. This was performed following the manufacturer’s instructions. Gal-9 mRNA probe was specifically designed by BioTechne’s team and we validated it on our samples making parallel staining with a positive (Mm-Ppib) and negative control (DapB) probe. Probes for CXCL9 and CXCL10 were catalog probes. After the signal detection step, slides were counterstained with 50% hematoxylin staining solution and dehydrated with 70% ethanol. After mounting slides, images were acquired with a Virtual Slide microscope VS120-SL (Olympus, Tokyo, Japan), 20X air objective (0.75 NA, 345 nm/pixel).

### Deep profiling of the expressed TCR beta repertoire

RNAs extracted from tumor fragments (as explained above) were used to profile the TCR beta repertoire of tumor lymphocytes. Wet lab and bioinformatics analyses were performed by iRepertoire Inc. (Alabama, USA). Sequencing libraries were generated using their proprietary “dimer avoiding multiplex” (dam)-PCR technology which uses iterative rounds of amplification and amplicon recovery to selectively amplify the TCR beta chains (all possible murine VDJ combinations in one single reaction). Libraries were then sequenced on an Illumina MiSeq platform and paired-end reads were generated. Data analysis and “tree map” illustrations were performed by iRepertoire Inc.

### Cytokines profiling

Protein extraction was performed from 30 mg of frozen tumor fragments by crushing them with TissueRuptor (Qiagen) in low detergent lysis buffer (20 mM Tric-HCl pH 7.5, 0.5% tween20, 150 mM NaCl) supplemented with the cOmplete Protease Inhibitor Cocktail (Roche Applied Science). Extracts were then clarified and protein concentrations were determined by the Bradford method using protein assay (Bio-Rad) and a microplate reader (Tecan Infinity F200 Pro). Undiluted samples from 1.23 to 5.38 mg/mL were used to perform the mouse cytokine array/chemokine array 31-Plex (MD31, Evetechnologies) (Eotaxin, G-CSF, GM-CSF, IFN-γ, IL-1α, IL-1β, IL-2, IL-3, IL-4, IL-5, IL-6, IL-7, IL-9, IL-10, IL-12 (p40), IL-12 (p70), IL-13, IL-15, IL-17A, IP-10, KC, LIF, MCP-1, M-CSF, MIG, MIP-1α, MIP-1β, MIP-2, RANTES, TNFα, VEGF). The cytokine concentrations were then normalized to protein concentrations.

### CXCL10 ELISA assay

WT (MB49, Ctrl # 116 and 376) and KO (KO # 175, 345 and 377) cells were cultured in normal media supplemented or not with recombinant murine IFN-γ (#315–05, PeproTech): 0, 2, 5, 10 and 25 ng/mL. After 72 h of IFN-γ treatment, supernatants were collected and secreted CXCL10 was assayed with mouse CXCL10 ELISA kit (#DY466-05, #DY008, R&D Systems) according to the manufacturer’s instructions.

### Statistical analysis

Except for RNAseq data, statistical tests were performed using GraphPad Prism version 7.00 (GraphPad Prism, RRID:SCR_002798). Kaplan–Meier survival curves were generated and analyzed using the log-rank, Mantel-cox test. Significant differences when comparing two groups were determined by two-tails Mann–Whitney test. Significant differences when comparing three groups were determined by Krustal–Wallis test followed by Dunn’s multiple comparisons test. *P* value < 0.05 was considered as significant.

## Supplementary Information

Supplementary Information
