# Supplementary material for: Serial transplantation unmasks galectin-9 contribution to tumor immune escape in the MB49 murine model
Source: Sci Rep. 2021 Mar 4;11:5227. doi: 10.1038/s41598-021-84270-1 (PMC7933353; doi:10.1038/s41598-021-84270-1)
Supplement: Supplementary file 1 — Supplementary Information [file 41598_2021_84270_MOESM1_ESM.pdf]

Serial transplantation unmasks galectin-9 contribution to tumor immune escape in the MB49 murine model

Valentin Baloche[1], Julie Rivière[2], Thi Bao Tram Tran[1], Aurore Gelin[1], Olivia Bawa[3], Nicolas Signolle[3], M'boyba Khadija Diop[4], Philippe Dessen[4], Stéphanie Beq[5], Muriel David[5], Pierre Busson[1]

[1] CNRS, UMR 9018, Gustave Roussy and Université Paris-Saclay, 39 rue Camille Desmoulins, F-94805 Villejuif, France.

[2] Inserm, U1170, Gustave Roussy, 39 rue Camille Desmoulins, F-94805 Villejuif, France.

[3] Plateforme d'évaluation préclinique, Gustave Roussy, 39 rue Camille Desmoulins, F-94805 Villejuif, France.

[4] Plateforme de Bioinformatique, UMS AMMICA, Gustave Roussy, 39 rue Camille Desmoulins, F-94805 Villejuif, France.

[5] HiFiBiO Therapeutics, Pépinière Paris Santé Cochin, 29 rue du Faubourg Saint-Jacques, 75014 Paris, France.

Running Title: Galectin-9 ablation and serial transplantation in the MB49 murine tumor model

Key words: Galectin-9, murine tumor models, serial transplantation, CRISPR/Cas9 gene editing, interferon-γ, CXCL10

Legends of Supplementary Figures

Supplementary Figure S1. Invalidation of the gal-9 gene in clones derived from the MB49 cell line. To knock-out the *Lgals9* gene using the CRISPR/Cas9 method, two guides were designed, inserted in plasmids expressing GFP or mCherry respectively and transfected simultaneously together with a Cas9 expressing construct in order to delete a sequence containing the gal-9 ATG start codon. Two days after transfection, single cells with concomitant high GFP and m-Cherry expression were sorted, seeded at one cell per well and grown in 96-well plates. After an average of 2 weeks of expansion, replicas of these clones were subjected to detection of intra-cellular gal-9 by flow cytometry. Using this procedure, we selected three gal-9-KO clones (KO #175, #345 and #377) and two WT-control clones (Ctrl #116 and #376). Control clones were derived from cells subjected to the same transfection and clonal selection processes, but which had retained gal-9 expression with an intact gene sequence. As the single cell growth step resulted in clonal growth of no more than 45% of the cells, the WT-Ctrl clones were essential to take into account the clonal selection process. (A) Schematic representation of the gal-9 gene (*Lgals9*) and the target sites of the CRISPR guides 1 and 2 located respectively in the first exon and the first intron. (B) Sequence analysis of the WT-Ctrl clones #116 and #376 indicated that the *Lgals9* locus were unaffected in both alleles (possible explanations might be absence of Cas9-mediated double-strand DNA break or efficient repair of the breaks). For the three-selected gal-9-KO clones, the deleted fragments were either replaced by a new DNA sequence (#377) or by the deleted fragments oriented in the reverse direction (#175 and #345). (C) Western-Blot analysis of the gal9 protein in the selected clones: the parental line (MB49) and the WT-Ctrl clones expressed two isoforms: gal-9L and gal-9M; the latter being predominant. The absence of Gal9 expression was confirmed in the gal-9-KO clones. Related full blots are displayed in the Supplementary Figure S9.

Supplementary Figure S2. *In vitro* growth of MB49 cells and isogenic clones (WT-Ctrl and gal-9-KO). The growth assays were performed using an ATP luminescence assay (ATPlite, Perkin Elmer). No differences in growth rate were

recorded *in vitro* between WT and KO cells. MB49 cells had doubling time of about 24h.

Supplementary Figure S3. *In vitro* transcriptional profiles of MB49 cells and isogenic clones (WT-Ctrl and gal-9-KO). For each cell type, the RNAseq analysis was performed on RNAs extracted from cells propagated *in vitro* including the parental cells, 2 WT-Ctrl clones and 3 gal-9-KO clones. (A) The volcano plots intend to represent genes differentially expressed when comparing gal-9-KO cells on the one hand with parental and WT-Ctrl cells on the other hand. They are virtually absent, except *lgals9.* (B) Results of the non-supervised hierarchical clustering are consistent with data from the volcano plots since the transcription profiles of gal-9-KO cells are not separated from those of the WT cells. Potential weak differences related to gal-9 invalidation seem to be outweighed by independent clonal differences.

Supplementary Figure S4. Transcriptional status of genes predicted to be vulnerable to off-target effects in the isogenic clones. A total of 9 exons (4 for the guide 1 and 5 for the guide 2) were identified as potential off-targets (**see Supplementary Table S2**). The abundance of the corresponding transcripts reflected by FPKM (fragments per kilobase of exon per million reads mapped) values were checked in the RNAseq data obtained from MB49 and its derived clones (either gal-9-KO or WT-Ctrl) grown *in vitro.* No difference was detected for any of these transcripts between parental cells and clones subjected to the CRISPR procedure.

Supplementary Figure S5. Comparative assessment of galectin-1, -3 and -8 transcription and protein expression in WT and gal-9-KO cells. (A) Abundance of galectin transcripts recorded by RNA-seq in parental MB49 and its derived clones. In blue, WT, parental or control clones. In red, gal-9-KO clones. (B) WB analysis were performed on the same cellular materials. All cells express galectins -1, -3, and -8 with the first two being the most abundant. The gal-9 invalidation has no impact on the expression of other galectins at both the transcriptional and the protein level. Related full blots are displayed in the Supplementary Figures S10 and S11.

Supplementary Figure S6. Schematic representation of the serial transplantation assay and recapitulation of biological investigations performed on MB49 cells and the related clones in syngenic mice. The mice were of the C57BL/6N strain. Each square represents a distinct mouse bearing a tumor derived from MB 49 parental cells, control clones (#116 and 376) or gal-9-KO-clones (#175, 345 and 377). The first inoculation resulting in the first cycle of tumor growth was done by subcutaneous injections of suspensions of cells grown *in vitro* (5.10^5 cells suspended in 100µL of PBS). Transplantations leading to the next cycles of growth were made by sub-cutaneous insertions of aggregates of tumor fragments (70 mg) collected from the previous cycle after 10 days of tumor growth. In most cases, tumor fragments collected from one animal were inoculated in a single recipient mouse. Thus, we propagated 3 tumor “lineages” for each gal-9-KO clones. For control clones and MB49 parental cells, only 2 “lineages” were propagated beyond the 2nd cycle because all of them were obviously very aggressive. One lineage of MB49 parental tumors was lost during the 3rd cycle because of the accidental death of one mouse. Out of 9 tumor “lineages” derived from gal-9-KO clones, five stopped their growth at cycle 4. Only one of them went beyond cycle 5. In contrast, all 5 evaluable tumor “lineages” derived from parental cells or control clones reach the 6th cycle, still being very aggressive. Symbols inside the squares represent the various types of biological investigations done on each tumor: immunohistochemistry for detection of leucocytes, especially CD4 and CD8 T-cells, and *in situ* detection of CXCL9 and CXCL10 mRNAs for some of them (IHC); deep profiling of expressed TCR-β repertoire (TCR-β); RNA-seq analysis (RNAseq); mRNA quantitation by RT-qPCR for genes encoding cytokines and MHC II components regulated by interferon-γ (qPCR); cytokine detection in tumor protein extracts (cytokines).

Supplementary Figure S7. **Details on tumor growth of various types of MB49 cells grafted on syngenic and nude mice.** (A) Growth curves for single WT tumors grafted on C57BL/6 mice are shown in blue. At the beginning of the experiment, each cell type - MB49 cells and WT-Ctrl clones - was injected subcutaneously into two mice (MB49: ▲,▼; WT-Ctrl #116: ■, ◆; #376: ●, ⬣). For the subsequent cycles of

tumor growth, each tumor was passaged on a single mouse. For a given tumor « lineage », the same symbol is used through the 4 cycles of tumor growth (B) Growth curves for KO tumors on C57BL/6 mice are shown in red (gal-9-KO clone #175: ▲,▼; #345: ■, ◆; #377: ●, ⬣). Again, for a given tumor « lineage », the same symbol is used through the 4 cycles. (C) From cycle 3, the growth of some KO tumors was not sufficient in the interval of 10 days to allow their subsequent passage on another mouse for an additional cycle of tumor growth (< 90mg). The consequence was the termination of this tumor « lineage ». To give a quantitative assessment of this aspect of tumor growth reduction, the survival of WT and KO tumor « lineages » are represented on a Kaplan-Meier plot (with blue and red color respectively). We observe that after 5 cycles of tumor growth, all WT tumor « lineages » are still active in contrast with only one for the KO tumors. (D and E) Growth curves for single WT (blue) and KO (red) tumors grafted into nude mice with the same mode of representation as for C57BL/6. (F) and (G) Comparative assessment of tumor weights at the termination of each cycle of tumor growth for WT (F) or KO (G) tumors grafted either into syngenic or nude mice. In the case of WT tumors (blue dots), there were no significant differences at cycle 2, 3 and 4. Regarding gal-9-KO tumors (red dots)b, the average tumor mass is consistently greater on the nude mice.

Supplementary Figure S8. Unsupervised RNAseq analysis of WT and KO tumors at different cycles of tumor growth. Dendrogram built using centered correlation and ward linkage.

Supplementary Figure S9. Full blot display for WB detection of gal-9 in MB49 clones and parental cells. Annex to Supplementary Figure S1C. The relevant blots are surrounded by a red dotted line.

Supplementary Figure S10. Full blot display for WB detection of galectin-1, -3, -8 in MB49 clones and parental cells. Annex to Supplementary Figure S5B. The relevant blots are surrounded by a red dotted line.

Supplementary Figure S11. Full blot display for WB detection of actin used as a loading control in MB49 clones and parental cells. Annex to Supplementary Figure S5B. The relevant blot is surrounded by a red dotted line.

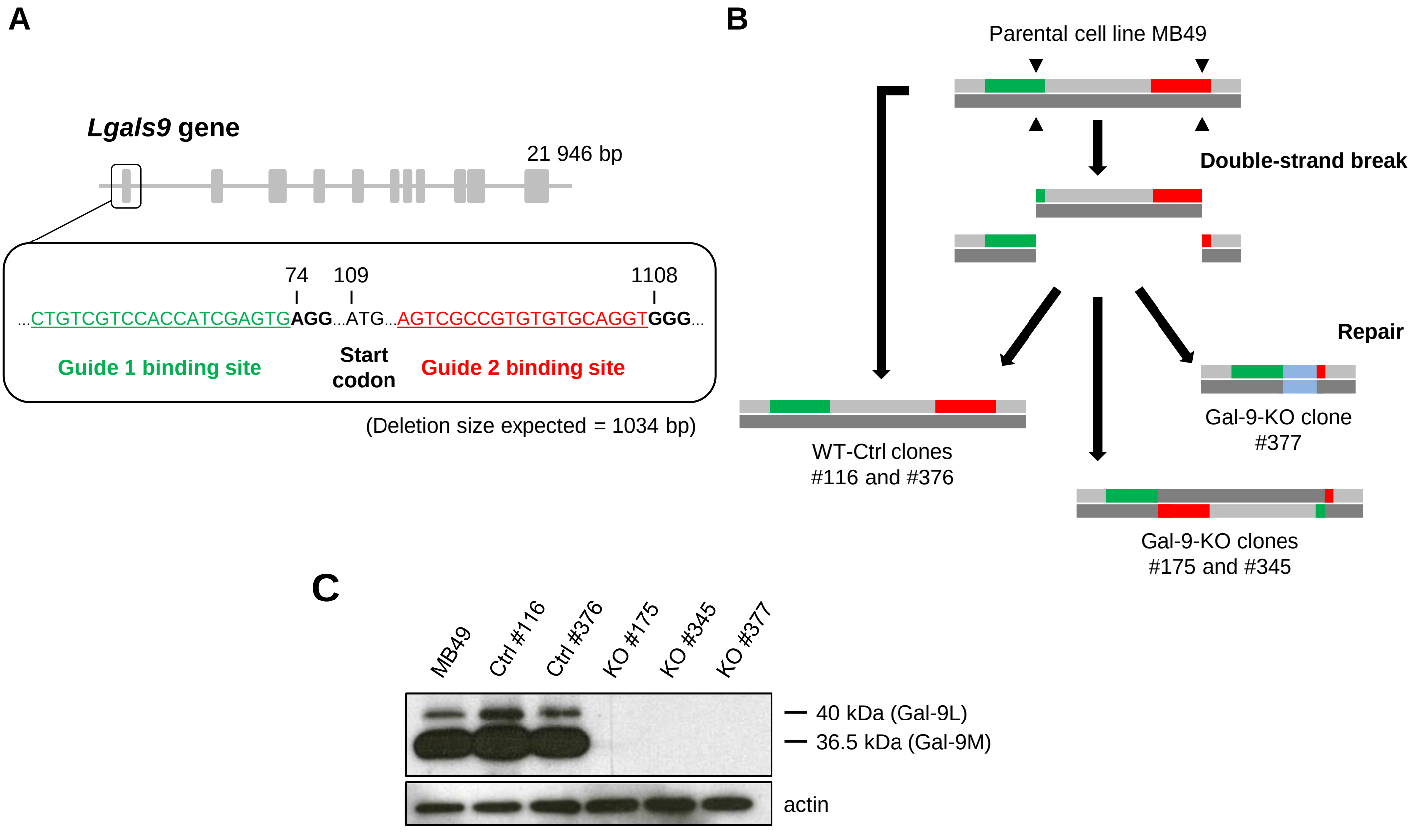

Supplementary Figure S1

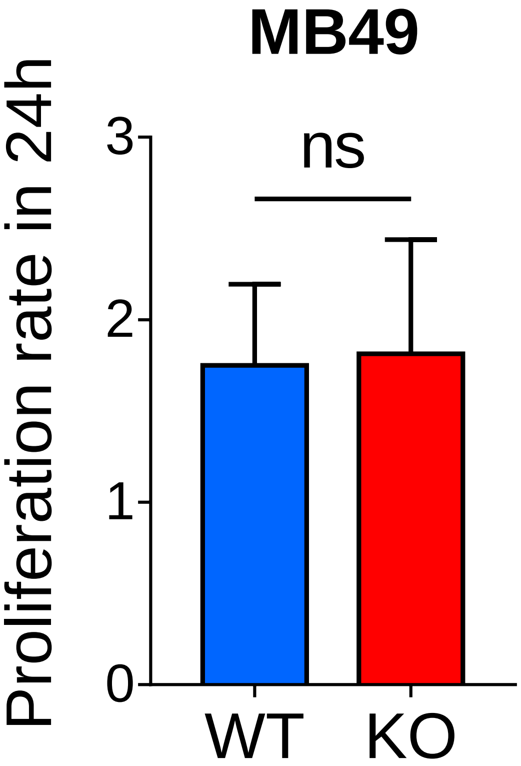

Supplementary Figure S2

A

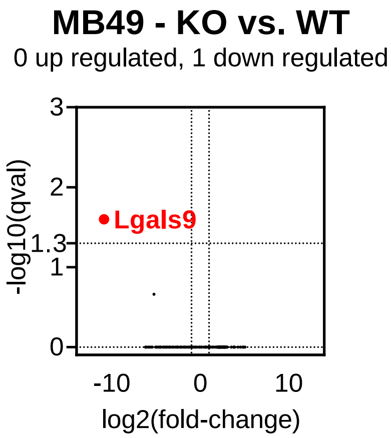

B

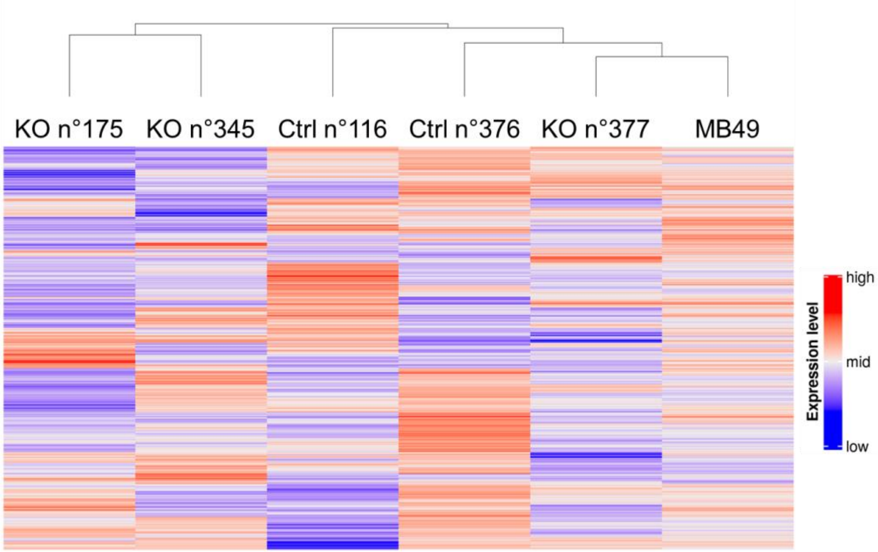

Supplementary Figure S3

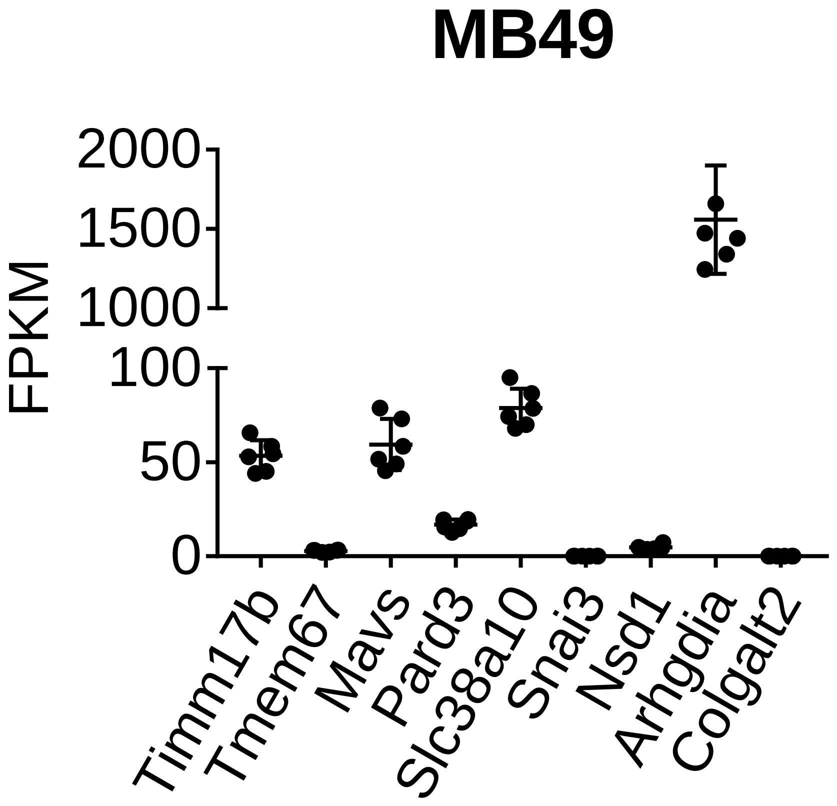

Supplementary Figure S4

A

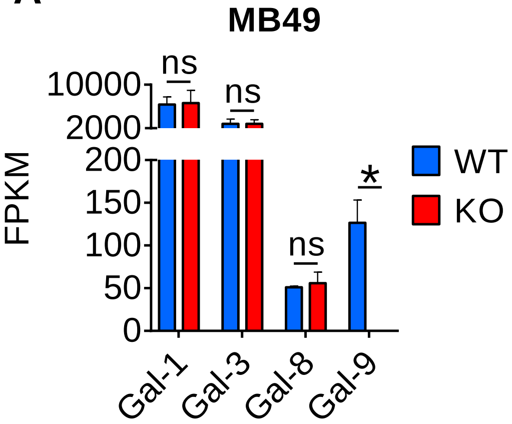

B

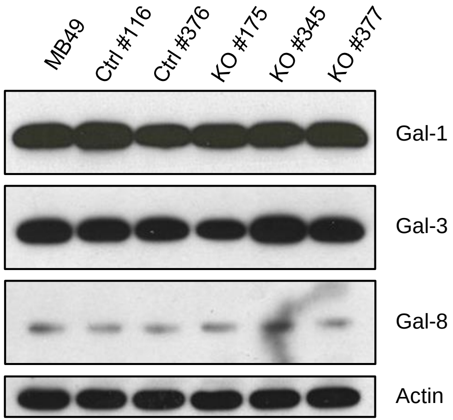

Supplementary Figure S5

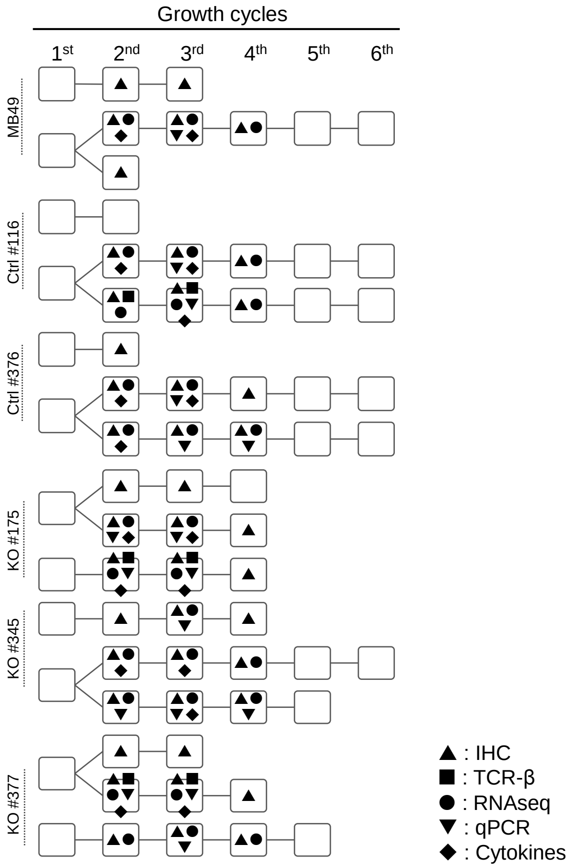

Supplementary Figure S6

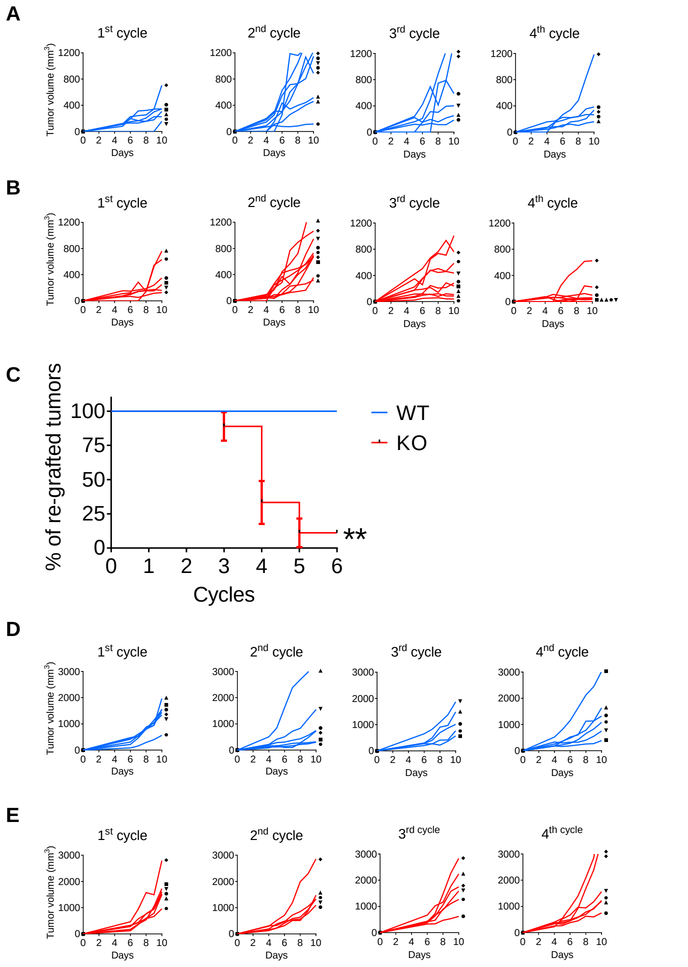

Supplementary Figure S7

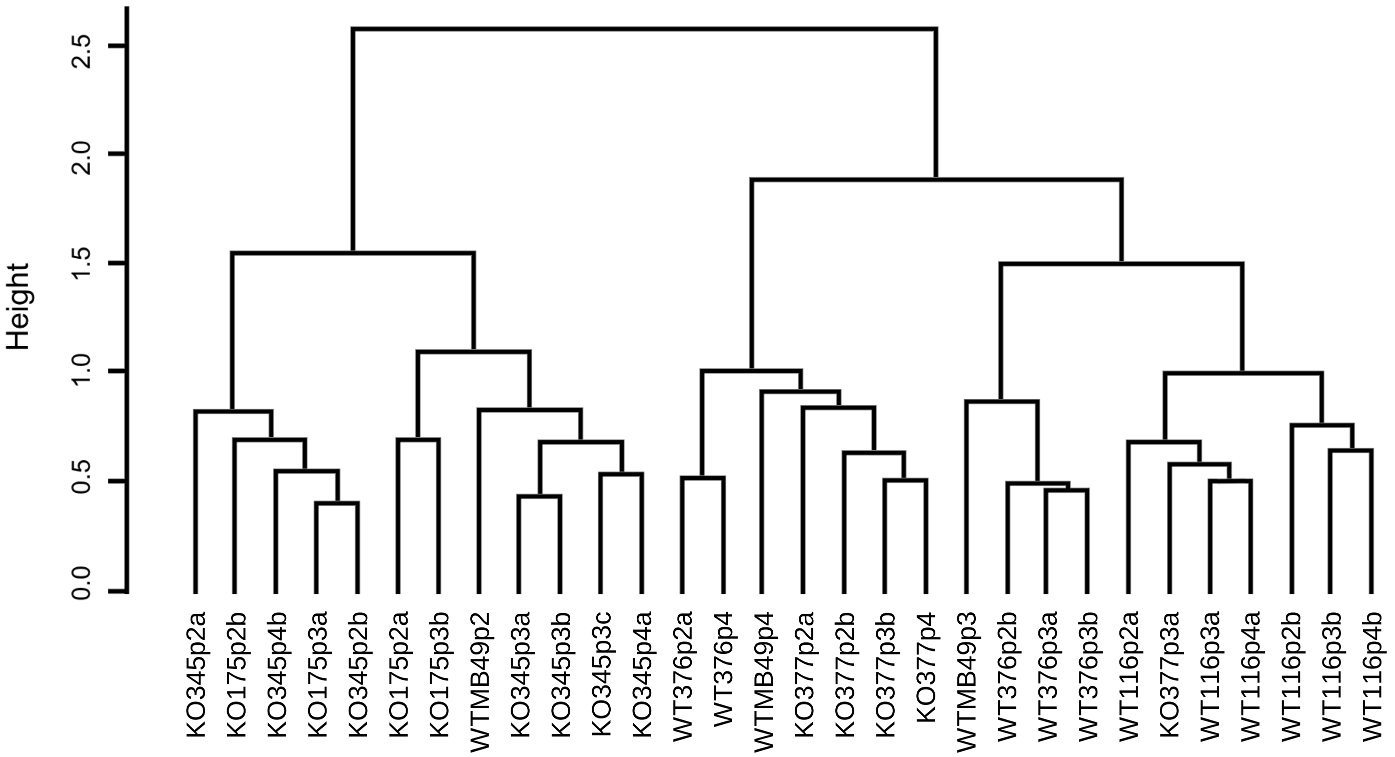

Supplementary Figure S8

Western blots for galectin-9 – Full blot display
Annex to Supplementary Figure S1C

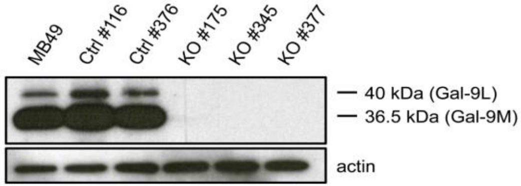

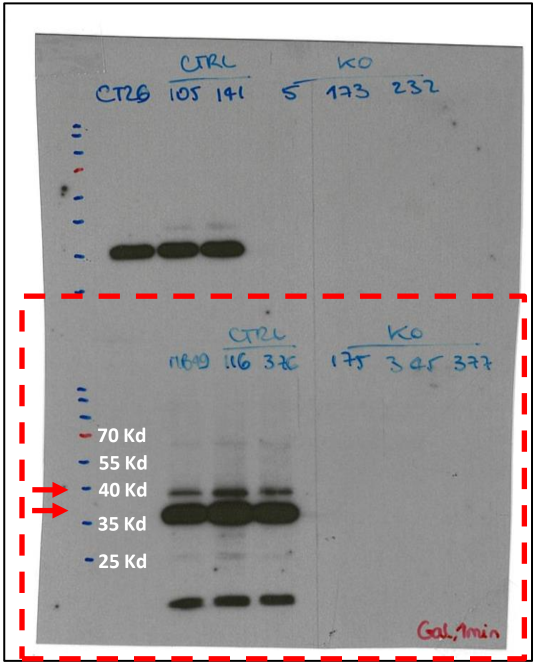

Galectin-9 (1min)

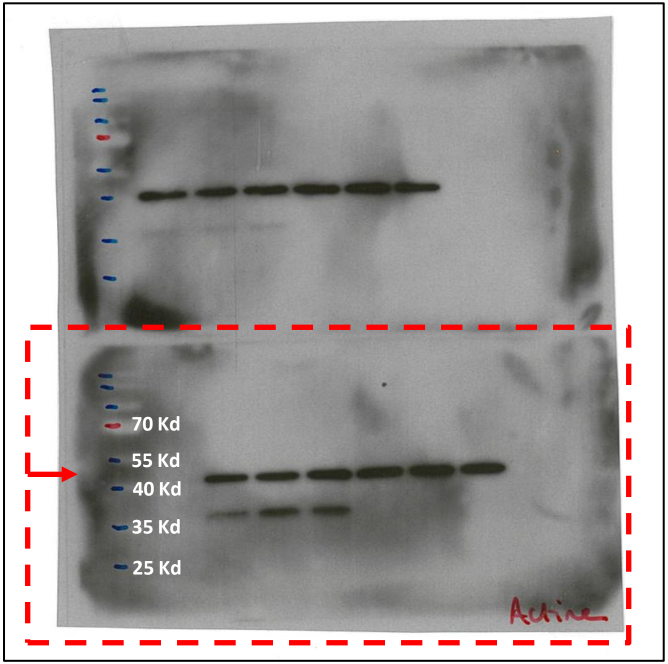

Actin (1min)

Supplementary Figure S9

Western blots for galectin-1, -3, -8 Full blot display
Annex to Supplementary Figure S5B

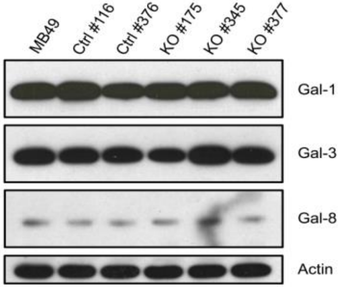

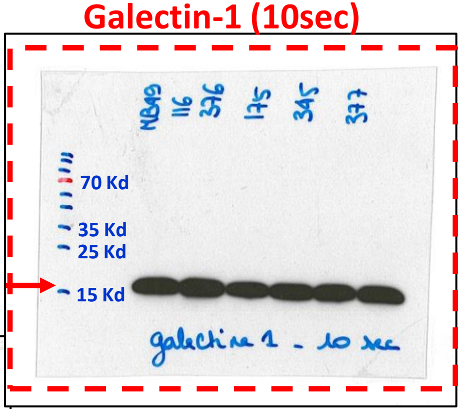

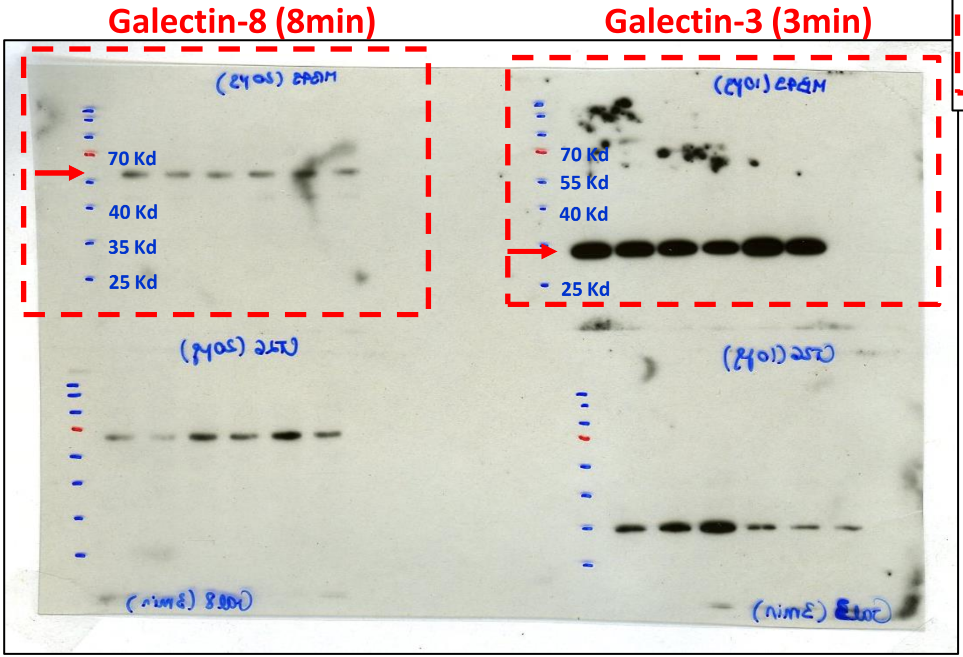

Supplementary Figure S10

Western blots for actin used as a loading control for western blots of Supplementary Figure S10
Full blot display
Annex to Supplementary Figure S5B

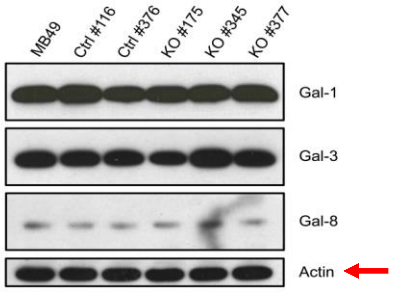

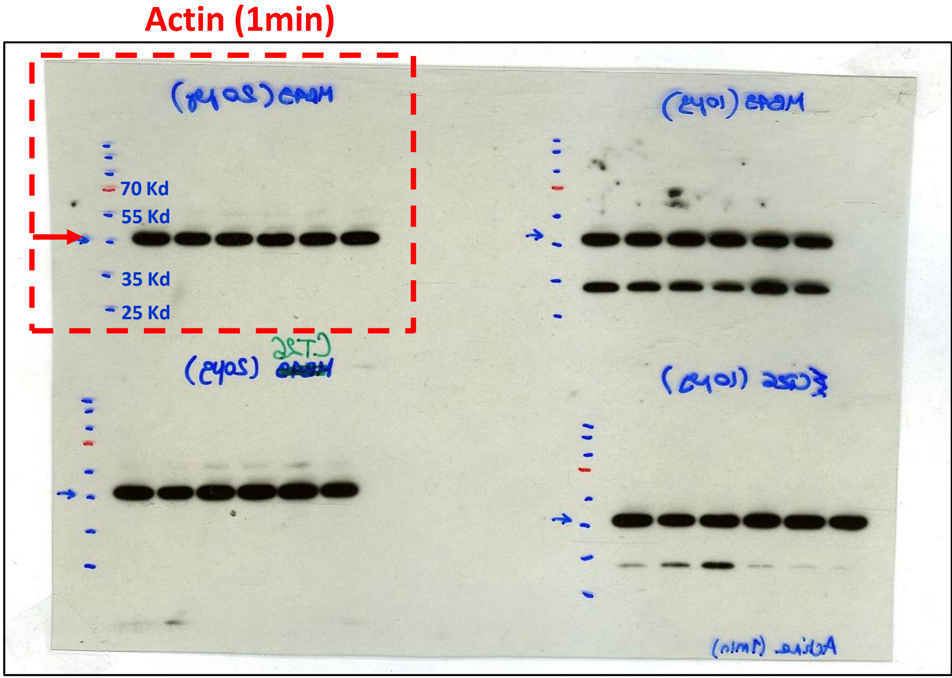

Supplementary Figure S11

Supplementary Table S1. Number of distinct sequences identified in various tumor samples by deep-profiling of TCR-β chain (CDR3) (1).

Samples	Total read number	Distinct sequences (clonotypes)	
		At least 1 read	> 100 reads
WT-Ctrl clone #116 (2nd cycle)<br>WT-Ctrl clone #116 (3rd cycle)	669,565<br>567,525	517<br>641 (+24%)	107<br>130 (+21%)
Gal-9-KO clone #377 (2nd cycle)<br>Gal-9-KO clone #377 (3rd cycle)	536,629<br>439,445	660<br>927 (+40%)	139<br>212 (+53%)
Gal-9-KO clone #175 (2nd cycle)<br>Gal-9-KO clone #175 (3rd cycle)	501,260<br>554,846	1,337<br>1,552 (+16%)	305<br>400 (+31%)

(1) The number of distinct sequences represented by at least one read is greater at cycle 3 than cycle 2 and for gal-9-KO than for WT tumors. These differences are more obvious if one looks at the distinct sequences represented by at least 100 reads. Their frequence reflects the abundance of clonotypes of average size represented by small rounded rectangles in Fig. 4.

Supplementary Table S2. Potential off-targets of each guide used in our genome editing procedure according to the CRISPOR web tool (1).

Guide 1	Guide 2
3:intergenic:Rnf168-Ubxn7	2:exon:Slc38a10
4:intron:Ahcyl2	4:intergenic:Fam173b-Tas2r119
3:exon:Timm17b	4:intergenic:Sec16b-Brinp2
3:intergenic:Ly96-Gm5828	4:intergenic:Gm10398-Gm26660
4:intergenic:Gm23274-Gm26901	3:intergenic:Cdh10-Gm25976
4:intergenic:Gm3867-Gm7257	4:intergenic:Gm7361-4930584F24Rik
4:intron:Dach1	4:intergenic:Mthfsd-Foxc2
4:intergenic:Gm22544-Gm22509	4:intergenic:Trpc6-Pgr
4:exon:Tmem67	4:intron:Kazn
4:intron:Banf2	4:intergenic:4933428C19Rik-Gm12391
4:intron:Zfp457	4:intergenic:Gm23905-Zfp36l2
4:intergenic:Gm10247-Grin2a	4:intergenic:S100z-F2rl1
4:intergenic:ZFP953	4:intergenic:Gm23406-Mis18a
4:intergenic:A330102I10Rik-Gm11363	4:intergenic:Gm25608-Pgm1
4:intergenic:Gm20918-Gm21820	4:intron:Cacna2d2
4:exon:Mavs	4:intergenic:Gm22016-Cdc73
4:intergenic:Gm23558-Galc	4:intron:Gm5155
4:intron:Mast4	4:intron:Gsg1l
4:intergenic:Rgs17-Gm10945	4:intergenic:Gm7452-1700111E14Rik
4:intron:Dst	4:intergenic:Nptx2-Gm6272
4:intergenic:Tmem108-Nphp3	4:exon:Snai3
4:intron:Dock8	4:intergenic:Kif26a-A730018C14Rik
4:intron:Col22a1	4:intergenic:Gm26674/Sash1-Gm9930
4:intergenic:Six2-Srbd1	4:intron:Car5a
4:intergenic:Dusp10-Gm23349	4:intron:Lpcat1
4:intergenic:Pkp1-Igfn1	4:exon:Nsd1
4:intergenic:Gm11468-Gm14268	4:exon:Arhgdia
4:intergenic:Prkaa2-Ppap2b	4:intron:Susd1
4:intron:Plekha7	4:intergenic:Gm13605-Gsn
4:intron:Mad1l1	4:intergenic:Fopnl-Abcc1
4:intergenic:Maf/Gm15655-Dynlrb2	4:intergenic:Gm5415-Gm24732
4:intron:Tspan32	4:intergenic:Gm20711-Pbx1
4:intergenic:1810011O10Rik/Gm26714-Ido2	4:intergenic:Carns1-Tbc1d10c
4:intergenic:Impg1-RP24-325L16.1	3:intergenic:2610028E06Rik-Csf3r
4:intron:Fam172a	3:intergenic:Gm26216-Trerf1
4:exon:Pard3	4:intron:Ccbl2
	4:intron:9030624J02Rik
	4:intergenic:Tex14-Gm11492
	4:intergenic:4931417E11Rik-Gm26179
	4:intergenic:A930003A15Rik/AC164558.1-Klhl6
	4:intergenic:Gm16202-Ube2cbp
	4:intergenic:Mitf-Gm765
	4:intron:Pex14
	4:exon:Colgalt2
	4:intergenic:Gm22132-Nr5a2
	4:intron:Filip1l
	3:intergenic:Rbfox1-Gm23476
	4:intron:Dpp10
	4:intron:Tns3
	4:intron:Ppard
	4:intergenic:Ntm-Gm26435
	3:intergenic:Gm25830-4930555F03Rik
	4:intergenic:Top1mt-Rhpn1
	4:intergenic:Hcfc1-Gm8545
	4:intron:Dcdc2a
	4:intron:Tenm3
	3:intron:Orc6
	4:intron:Sh3pxd2a
	4:intron:Wdtc1
	4:intron:Ift122
	4:intergenic:Gm12718-Gm23064
	4:intergenic:Gm9855-Foxo3

(1) The CRISPOR web tool was applied on the mm10 Mouse genome. The number preceding the name of each genome locus indicates the number of mismatches required for complete alignment of the guide sequence. For both guides, none of the predicted target sites shows a perfect match.
